# Molecular basis of the CYFIP2 and NCKAP1 autism‐linked variants in the WAVE regulatory complex

**DOI:** 10.1002/pro.5238

**Published:** 2024-12-11

**Authors:** Song Xie, Ke Zuo, Silvia De Rubeis, Paolo Ruggerone, Paolo Carloni

**Affiliations:** ^1^ Computational Biomedicine Institute of Advanced Simulation IAS‐5 and Institute of Neuroscience and Medicine INM‐9, Forschungszentrum Jülich GmbH Jülich Germany; ^2^ Department of Physics RWTH Aachen University Aachen Germany; ^3^ National & Local Joint Engineering Research Center of Targeted and Innovative Therapeutics, Chongqing Key Laboratory of Kinase Modulators as Innovative Medicine College of Pharmacy (International Academy of Targeted Therapeutics and Innovation), Chongqing University of Arts and Sciences Chongqing China; ^4^ Department of Physics University of Cagliari Monserrato Cagliari Italy; ^5^ Seaver Autism Center for Research and Treatment Icahn School of Medicine at Mount Sinai New York New York USA; ^6^ Department of Psychiatry Icahn School of Medicine at Mount Sinai New York New York USA; ^7^ The Mindich Child Health and Development Institute Icahn School of Medicine at Mount Sinai New York New York USA; ^8^ Friedman Brain Institute Icahn School of Medicine at Mount Sinai New York New York USA; ^9^ JARA Institute: Molecular Neuroscience and Imaging Institute of Neuroscience and Medicine INM‐11, Forschungszentrum Jülich GmbH Jülich Germany

**Keywords:** autism‐linked mutations, hotspot analysis, protein isoforms, protein–protein docking, WAVE regulatory complex

## Abstract

The WAVE regulatory pentameric complex regulates actin remodeling. Two components of it (CYFIP2 and NCKAP1) are encoded by genes whose genetic mutations increase the risk for autism spectrum disorder (ASD) and related neurodevelopmental disorders. Here, we use a newly developed computational protocol and hotspot analysis to uncover the functional impact of these mutations at the interface of the correct isoforms of the two proteins into the complex. The mutations turn out to be located on the surfaces involving the largest number of hotspots of the complex. Most of them decrease the affinity of the proteins for the rest of the complex, but some have the opposite effect. The results are fully consistent with the available experimental data. The observed changes in the WAVE regulatory complex stability might impact on complex activation and ultimately play a role in the aberrant pathway of the complex, leading to the cell derangement associated with the disease.

## 1 INTRODUCTION

Autism spectrum disorder (ASD) is a neurodevelopmental disorder characterized by deficits in social interaction and communication and restricted or repetitive patterns of interests and behaviors. ASD has a strong genetic component (Gaugler et al. 2014; Willsey et al. 2022), and hundreds of new ASD risk genes have been discovered with the advent of genomics (De Rubeis et al. 2014; Fu et al. 2022; Iossifov et al. 2014; Satterstrom et al. 2020; Zhou et al. 2022).

Among the genetic loci that have been recently associated with increased ASD liability are *CYFIP2* (Cytoplasmic FMR1‐Interacting protein 2) and *NCKAP1* (Non‐Catalytic region of tyrosine Kinase Associated Protein 1 (Fu et al. 2022)). In fact, based on rare de novo heterozygous single‐nucleotide variation, *CYFIP2* and *NCKAP1* both meet exome‐wide significance for association with ASD (Fu et al. 2022). Further, de novo heterozygous missense mutations in *CYFIP2* have been related to a form of developmental and epileptic encephalopathy characterized by early‐onset intractable seizures, severe to profound developmental delay, hypotonia, and mild facial dysmorphisms (MIM #618008) (Nakashima et al. 2018; Zweier et al. 2019). De novo or inherited variants likely leading to *NCKAP1* haploinsufficiency have been reported in individuals with ASD or autistic features, language and motor delays, variable degrees of intelligence disability, or learning disabilities (Guo et al. 2020). These clinical associations are compatible with the observation that both genes are under significant genomic constraints, as evidenced by their intolerance to missense or loss‐of‐function genetic variance in the general population (Karczewski et al. 2021).

**FIGURE 1:**
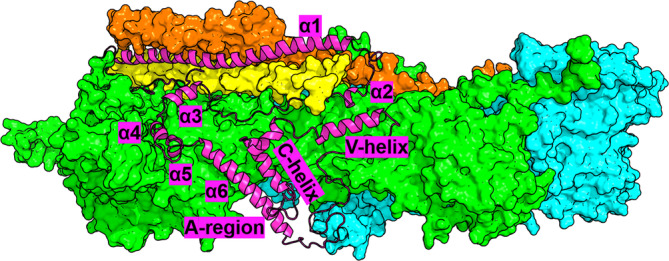
Structural schematic of WRC. CYFIP1/2, NCKAP1, ABI2, and HSPC300 are displayed as green, cyan, yellow, and pink surfaces, respectively. WAVE1 is highlighted with a magenta cartoon representation, with its structural details labeled. The model is built by SWISS‐MODEL (Waterhouse et al. 2018) and MODELLER V9.19 (Webb and Sali 2021) based on the crystal structure of the complex (PDB ID: 3P8C (Chen et al. 2010)).

The proteins encoded by *CYFIP2* (also known as PIR121) and *NCKAP1* (also known as NAP1) are part of a highly conserved hetero‐pentameric complex that regulates actin remodeling, the so‐called WAVE regulatory complex (WRC). The latter is formed by an elongated pseudo‐symmetric dimer formed by CYFIP1/2 and NCKAP1 (Chen et al. 2010) and then a trimer (Chen et al. 2010) consisting of the Abl interactor 2 (ABI2; or the orthologues ABI1 or ABI3), the hematopoietic stem progenitor cell 300 (HSPC300), and the Actin‐binding protein WASF1 (WAVE1; or the orthologues WAVE2 or WAVE3) (Rottner et al. 2021) (Figure 1). Combinations of different orthologs of each of these components can yield diverse WRC isoforms, likely with tissue, cellular, or developmental specificity. In particular, three CYFIP2 and two NCKAP1 isoforms are associated with ASD (CYFIP2_iso1_, CYFIP2_iso2_, and CYFIP2_iso3_ (a computationally mapped potential isoform (Consortium TU 2022); Table S1; NCKAP1_iso1_ and NCKAP1_iso2_, Table S2)).

WRC is critical for modulating actin remodeling: indeed, the VCA region of WAVE1 (WCA; Figure 1) binds to and activates the Arp2/3 complex to promote actin filament (Figure S1). In the basal state, the functional role of WCA is inhibited by interacting with CYFIP1/2 (Chen et al. 2010). When CYFIP1/2 binds two Rac1 proteins in specific sites (A‐site and D‐site in Figure S1a) (Chen et al. 2017; Schaks et al. 2018; Ding et al. 2022), it releases the WCA (“activation” process) (Figure S1). This process is essential for brain development and function, including the formation and maturation of synapses (De Rubeis et al. 2013; Hsiao et al. 2016; Oguro‐Ando et al. 2015; Pathania et al. 2014).

Studies have begun to examine the structural and functional impact of some disease‐associated mutations located in CYFIP2 or NCKAP1 (WAVE1, HSPC300, and ABI2 variants play no role in ASD (Fu et al. 2022); see Tables S1 and S2). The Arg87 (R87) of CYFIP2 is a mutational hotspot, and p.Arg87Cys/Ser/His/Pro/Lys pathogenic substitutions have been identified as associated with neurodevelopmental manifestations (Begemann et al. 2021; Zweier et al. 2019). R87 is located at the CYFIP2 interface with WAVE1 (Figure 1), as shown by an X‐ray structure of the entire WRC (Chen et al. 2010). The affinity of wild‐type (WT) CYFIP2 for WCA is larger than that of the R87C/L/P CYFIP2 ASD‐linked variants (Nakashima et al. 2018). Such high affinity is essential to keep WRC in an inactive state (Chen et al. 2010; Takenawa and Suetsugu 2007), and indeed, R87C (along with p.Ile664Met, p.Glu665Lys, p.Asp724His, and p.Gln725Arg, all of these ASD‐associated mutations involving residues located at the CYFIP2 interface with WAVE1 (Chen et al. 2010)) activate WRC abnormally, leading to aberrant lamellipodia (Schaks et al. 2020). These phenomena might be caused, at least in part, by a decrease in stability of the WAVE1/CYFIP2 interface (Figure 1) and/or by structural changes in the variants (Biembengut et al. 2022; Schaks et al. 2020). The p.Ala455Pro(CYFIP2) ASD‐linked variant leads to lamellipodia (Schaks et al. 2020). A455(CYFIP2) is very close to the CYFIP2/NCAKP1 interface (Chen et al. 2010). This may perturb the latter, perhaps via an allosteric mechanism (Schaks et al. 2020).

Here, we sought to systematically assess the structural impact of de novo missense mutations in CYFIP2 and NCKAP1 associated with ASD and co‐morbid neurodevelopmental manifestations in the assembly and function of the WRC, by prioritizing those hitting residues located at or closed to the protein/protein interface. These are almost all the mutations in CYFIP2 (14 out of 18, Tables 1 and S1), and very few in NCKAP1 (2 out of 15, Tables 1 and S2). For the first time, we map these disease‐linked mutations on the appropriate isoforms of CYFIP2 and NCKAP1 (Tables S1 and S2). It is essential to consider the different isoforms of the same protein because isoforms are diversely expressed in tissues. For instance, CYFIP2_iso2_ is mainly present in the human fetal brain (Patowary et al. 2024). First, we use a combination of homology modeling, blind protein–protein docking, all‐atom post‐docking refinement, and free energy‐based re‐ranking to predict the structural determinants of the WT WRC. The calculations are based on the X‐ray structure of the complex (Chen et al. 2010) and consider the correct isoforms for CYFIP2 and NCKAP1.^1^ Next, we perform a hotspot prediction on the predicted complexes to get qualitative insights on the relative stability of the eight protein/protein contact surfaces of the complex (Figure 1). Strikingly, we find that the ASD‐linked mutations in Table 1 affect the two interfaces of WRC with the largest number of hotspots by far, possibly impacting WRC stability. The impact of the ASD‐linked variants on the structural determinants of the complexes is finally investigated by our set of tools. These results could pave the way for a detailed blue map of WRC‐mediated biological pathways.

**TABLE 1:** ASD‐linked mutations of CYFIP2_iso1–3_ and NCKAP1_iso2_ involving positions at or close to the CYFIP2/WAVE1 and CYFIP2/NCKAP1 interfaces in the C1_iso1,WT_·WRC X‐ray structure (Chen et al. 2010).

CYFIP2	Mutation(s)	NCKAP1	Mutation
Isoform 1	**p.Arg87Cys/Leu/Pro**	Isoform 2	p.Pro372Thr
	**p.Ala455Pro**		
	**p.Ile664Met**		
	**p.Glu665Lys**		
	**p.Asp724His**		
	**p.Gln725Arg**		
Isoform 2	**p.Arg87Cys**		
Isoform 3	*p.Ile613Met*		**p.Arg675Thr**
	*p.Glu614Lys*		
	**p.Tyr639Cys**		
	*p.Gln674Arg*		
	**p.Arg744Cys**		

*Note*: The mutations that, according to our predictions (vide infra), turn out to weaken (strengthen) the interactions at the protein interface are colored in bold (italic). Several other variants (Tables S1 and S2) are located inside the proteins, and they are not investigated here.

## 2 RESULTS AND DISCUSSION

### 2.1 CYFIP2_iso1–_ _3_ ASD‐linked variants

We first predicted the structure of the WT complexes with the three isoforms of CYFIP2, and then, based on this structural information, we investigated the effect of mutations associated with ASD.

#### 2.1.1 Structural predictions of wild‐type CYFIP2_iso1–_ _3_·NCKAP1_iso1_ ·ABI2·HSPC300·WAVE1 complex (C2_iso1–3,WT_·WRC hereafter)

**FIGURE 2:**
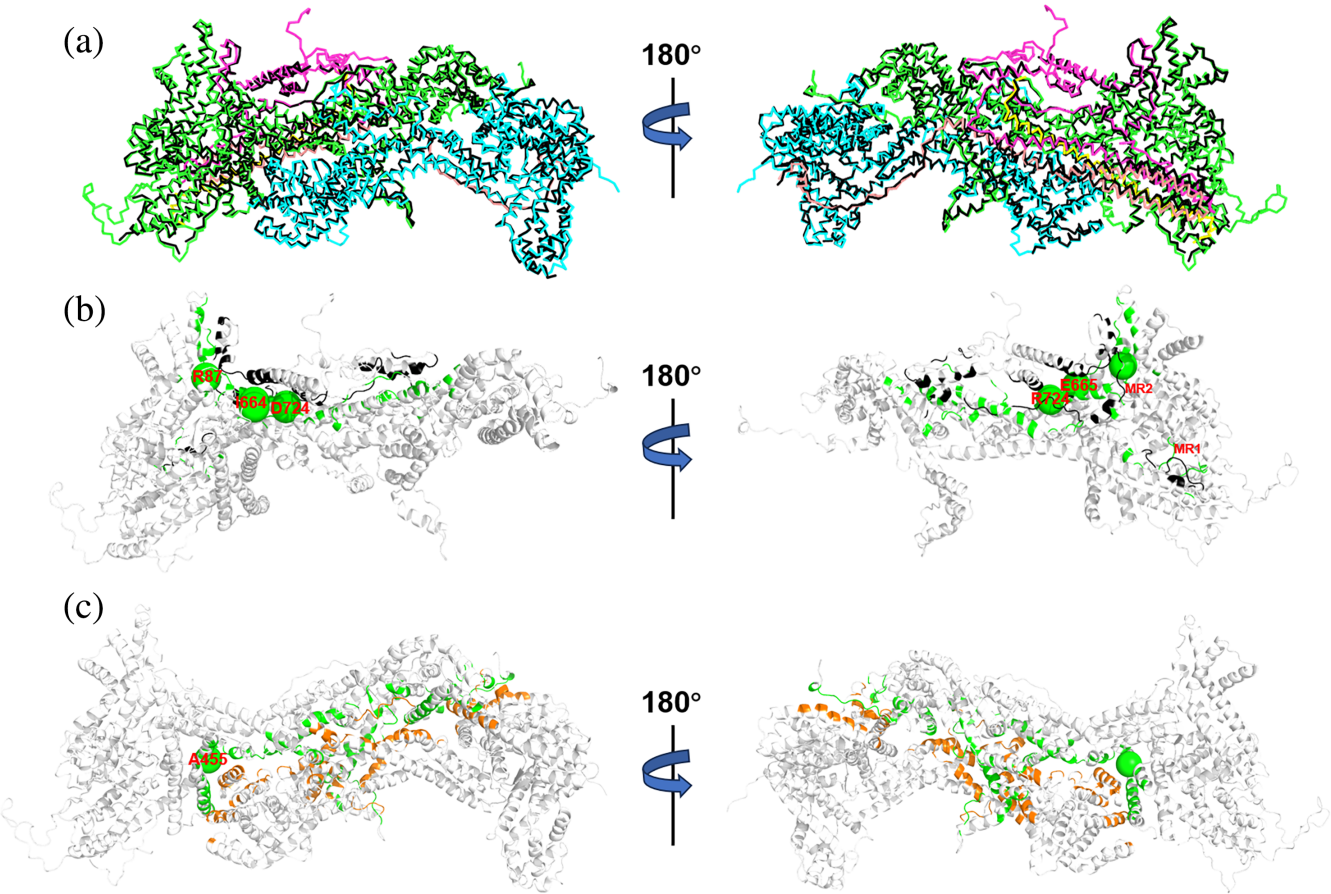
Structural prediction of C2_iso1,WT_·WRC. (a) Superposition of C1_iso1,WT_·WRC X‐ray structure (black Cα‐trace) with our C2_iso1,WT_·WRC structural model. The five components of the complex (CYFIP2_iso1_, NCKAP1_iso1_, WAVE1, HSP300, and ABI2) are shown in green, cyan, magenta, yellow, and pink Ca‐traces, respectively. Cartoon representation of the (b) WAVE1/CYFIP2_iso1_ and (c) NCKAP1_iso1_/CYFIP2_iso1_ contact surfaces, colored in black/green and orange/green, respectively. The Cα atoms of the residues undergoing ASD‐associated mutations are labeled and shown as larger for adjacent residues (I664, E665 and D724, Q725) and smaller spheres for the others. The MR1 and MR2 loops of CYFIP2_iso1_ are also labeled. The C2_iso2–3,WT_·WRC complexes have similar configurations (Figures S3 and S4).

We predicted the structural determinants of the three isoforms using homology modeling based on the WT CYFIP1_iso1_·NCKAP1_iso1_·ABI2·HSPC300·WAVE1 (C1_iso1,WT_·WRC hereafter) X‐ray structure (PDB ID: 3P8C (Chen et al. 2010)). The RMSD on the Cα atoms is 0.9 Å or less compared to the latter (Table S3), suggesting that the three CYFIP2 isoforms are accommodated in the complex similarly to CYFIP1_iso1_ (Figure 2a). Indeed, the isoforms differ only by the following: (i) The residues 558–582 (hereafter called MR1) are present only in CYFIP2_iso1_. They interact with WAVE1 N‐terminus. (ii) Residues 70–95 (hereafter called MR2) are present only in CYFIP2_iso1–2_. (iii) The first 50 residues of CYFIP2_iso3_ are unfolded. The sequence differences between CYFIP1_iso1_ and three CYFIP2 isoforms are shown in Figure S2a and Table S4. The structure of CYFIP1_iso1_ is the most similar to the CYFIP2_iso2_, without MR1 and having the MR2 (Figure 2a).

The three CYFIP2 isoforms interact with NCKAP1, WAVE1 (Figure 2b,c), HSPC300, ABI2 (Figures S2–S4 and Tables S5–S7) as CYFIP1_iso1_ does in the C1_iso1,WT_·WRC X‐ray structure (Chen et al. 2010) (Figures 1 and 2a). MR1 and MR2 are located at the CYFIP2/WAVE1 interface (Figure 2b).

**FIGURE 3:**
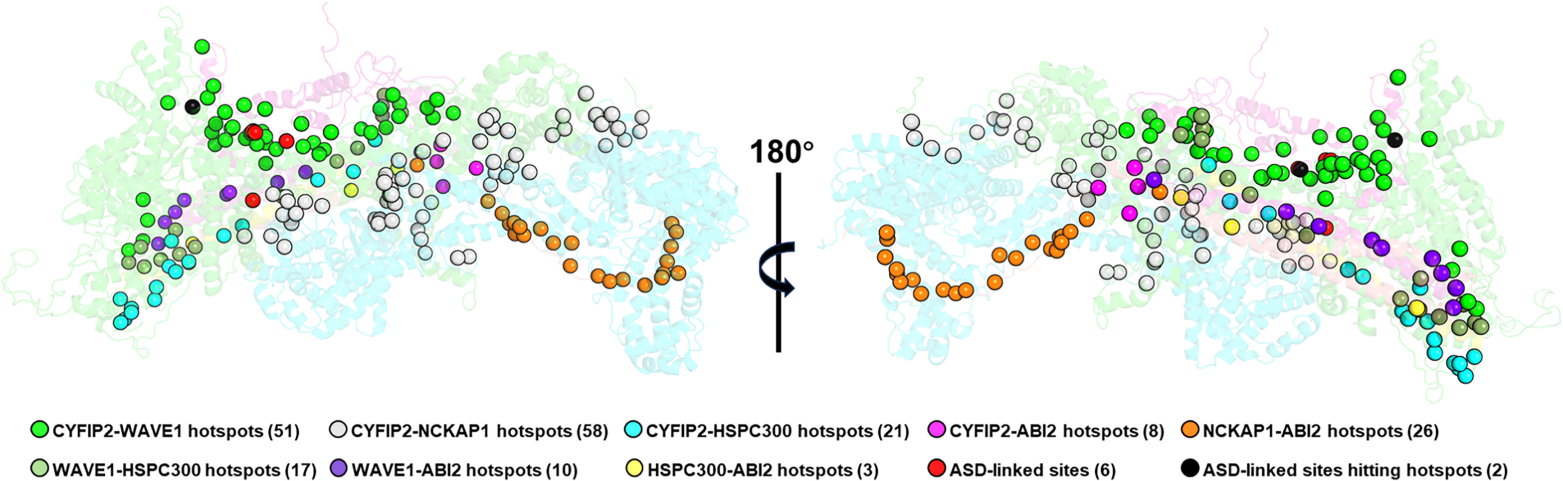
Hotspot predictions in C2_iso1,WT_·WRC using the mCSM server (Pires et al. 2013). The five components of the complex are shown as in Figure 2. The hotspots at the protein interfaces are shown as spheres of different colors. Two of them (R87 and D724) are also residues undergoing mutations in ASD (Table 1). Similar results are obtained using the BeAtMuSiC (Dehouck et al. 2013) and the FoldX (Schymkowitz et al. 2005) servers (Figures S6 and S7) and for the C2_iso2–3,WT_·WRC complexes (Figure S5).

The CYFIP2_iso1–3_/NCKAP1_iso1_ and CYFIP2_iso1–3_/WAVE1 feature the highest number of hotspots by far, irrespectively of the method used (Table 2 and Figures 3 and S5–S7), suggesting that quite tight interactions are formed at these interfaces. Thus, the ASD‐linked mutations of Table 1 perturb the two most critical protein/protein interactions of the complex in terms of hotspots, either directly by removing (R87(CYFIP2_iso1–2_), Q674(CYFIP2_iso3_), or D724(CYFIP2_iso1_), see Figures 3 and S5) or indirectly by altering the protein/protein interactions at the interfaces.

**TABLE 2:** Number of hotspots at all protein/protein interfaces of C2_iso1,WT_·WRC, C2_iso2,WT_·WRC, and C2_iso3,WT_·WRC, as predicted by the mCSM (Pires et al. 2013), BeAtMuSiC (Dehouck et al. 2013), and FoldX (Schymkowitz et al. 2005) servers (iso1/iso2/iso3).

Interfaces	mCSM	BeAtMuSiC	FoldX
CYFIP2/NCKAP1	58/54/60	47/53/54	93/95/111
CYFIP2/WAVE1	51/45/34	40/36/26	79/66/55
CYFIP2/HSPC300	21/23/19	11/9/10	24/15/19
CYFIP2/ABI2	8/7/10	8/6/6	16/9/13
NCKAP1/ABI2	26/19/23	11/8/11	16/26/18
WAVE1/HSPC300	17/13/12	14/15/11	40/33/33
WAVE1/ABI2	10/17/21	18/16/15	28/32/30
HSPC300/ABI2	3/4/5	11/11/10	21/21/18

#### 2.1.2 Structural predictions of C2_iso1–3_·WRC variants

**FIGURE 4:**
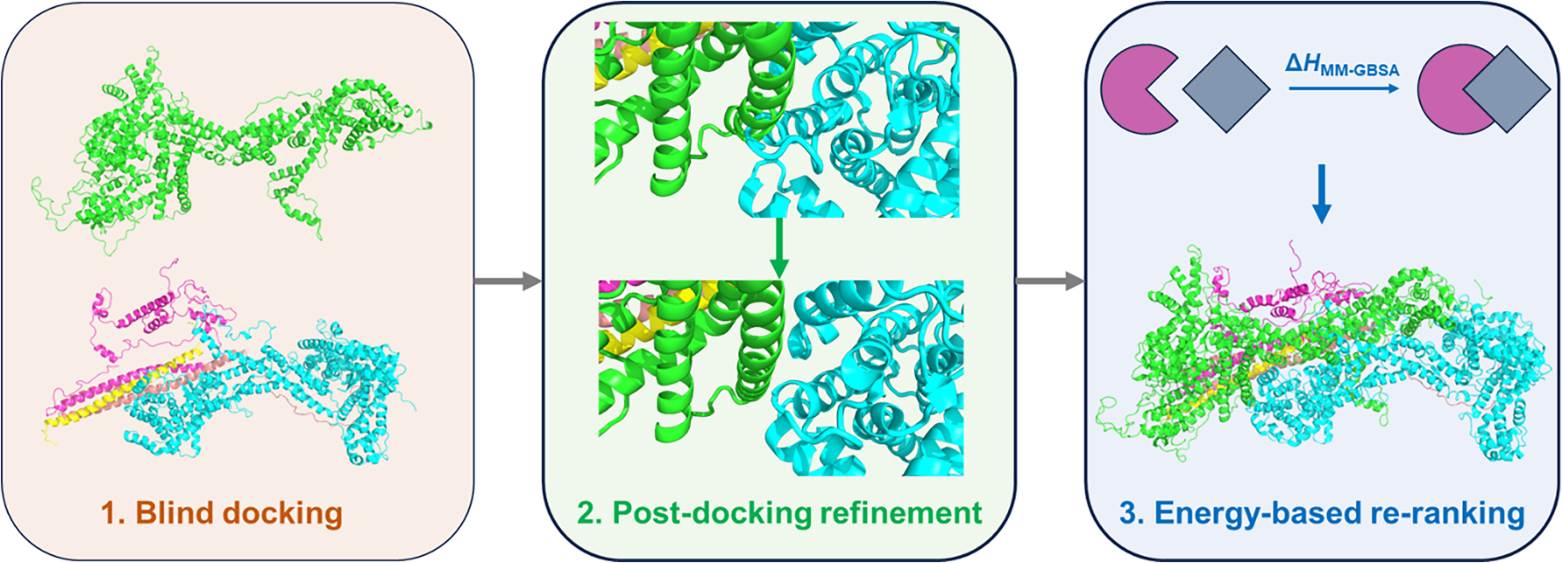
Our docking‐based protocol to predict the structural determinants of the variants in Table 1. The protocol turns out to reproduce accurately the structural determinants of C2_iso1–3,WT_·WRC starting from the CYFIP2_iso1–3_ and the rest of the complex, assessing the robustness of our protocol (see Data S1, Figure S8 and Table S3). Then, we applied our protocol to all the ASD‐linked variants at or close to the interface of CYFIP2_iso1–3_ (Table 1). Significant differences between using our protocol and simple homology modeling are observed (see Data S1 and Figure S9), indicating the importance of using our flexible approach for this system.

A quick way to model variants is to replace the amino acids at the protein/protein interface involved in the mutations. However, this approach does not consider the potential rearrangement of the interface caused by the mutation. This may be particularly important when dealing with homology models and not experimental structures (Fiser 2010). Here, to address this issue, we propose a docking‐based strategy by combining blind docking, refinement, and energy‐based re‐ranking (Figure 4). Specifically, we use the relative affinity index (see section 4 for a definition) to qualify the impact of mutations and isoforms on the stability of the complexes. Of course, this does not consider entropy effects and is used strictly for qualitative comparisons. If available, we report experimental findings on binding affinities.

##### CYFIP2_iso1_ /WAVE1 interface

For all the variants at the interface, the contact maps hardly change on passing from the WT to the ASD‐linked variants (Figures S10–S13), except, of course, the region of the mutations.

**FIGURE 5:**
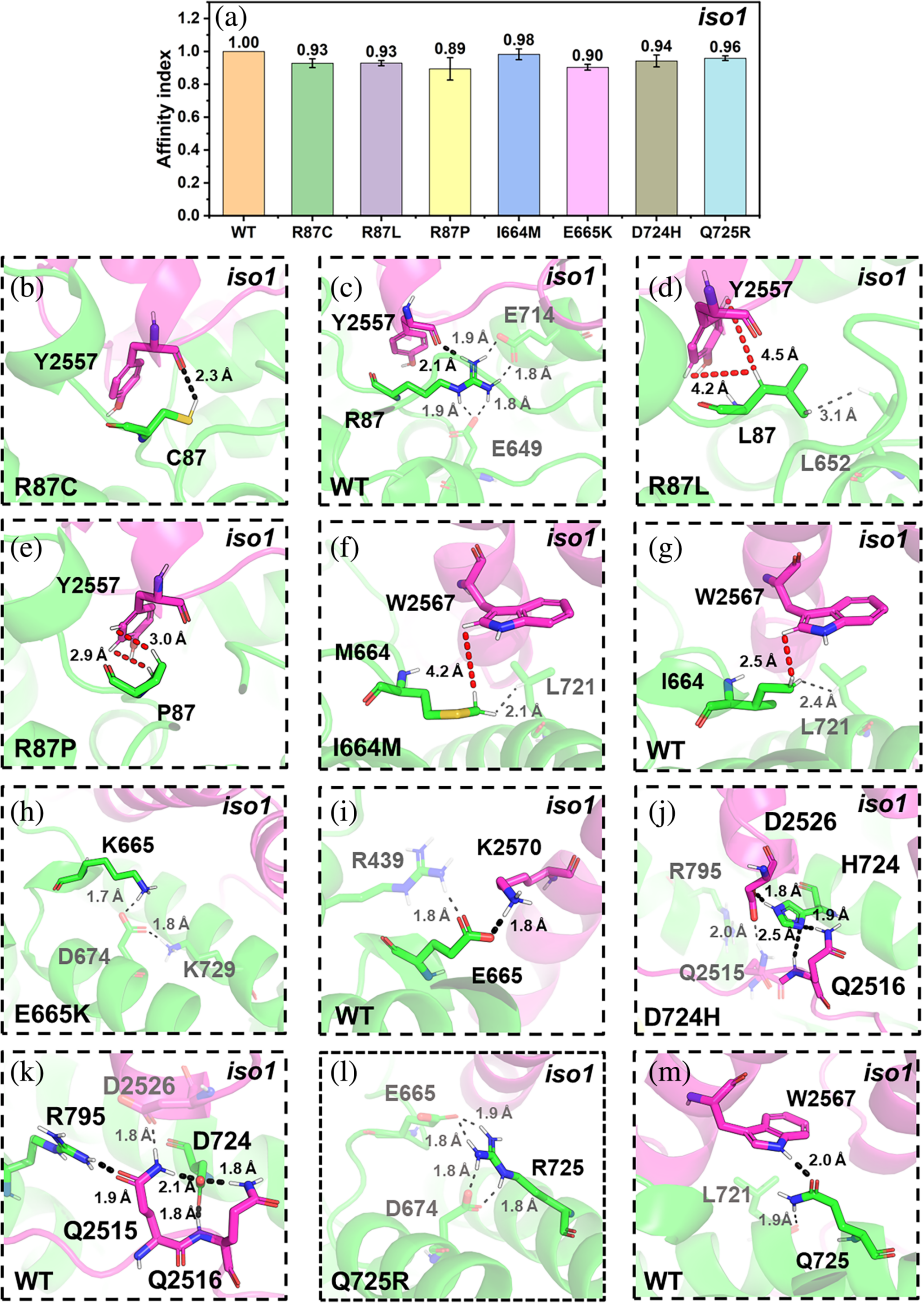
ASD‐related mutations at the CYFIP2_iso1_/WAVE 1 interface. (a) The affinity index between CYFIP2_iso1_ and WAVE1 in C2_iso1,WT_·WRC and the corresponding variants. The values are represented as mean ± SD (*N* = 3). (b–m) Alterations associated with the R87C/L/P (b–e), I664M (f, g), E665K (h, i), D724H (j, k), and Q725R (l, m) mutations. The CYFIP2_iso1_ and WAVE1 backbones are shown in Figure 2. Selected residues at the interfaces are represented by sticks. Only hydrogen atoms bound to polar groups or participating in hydrophobic interactions are shown. H‐bonds and van der Waals interactions are represented as black and red dashed lines, respectively.

**C2**_**iso1,R87C**_ **·WRC**: C87(CYFIP2_iso1_) forms a weaker hydrogen bond with Y2557(WAVE1) (Figure 5b) than that formed by R87(CYFIP2_iso1_), a hotspot, with the same residue in the WT complex. These observations are consistent with the decrease in experimental binding affinity (Nakashima et al. 2018) and in the calculated affinity index between the two proteins on passing from the WT to the mutant (Figure 5a). As a functional region of WAVE1, the disrupted binding between WCA and CYFIP2 could activate the WRC (Schaks et al. 2018). Moreover, decreased interactions between the Stem region/α‐6 helix of WAVE1 and CYFIP2 have a similar effect on WRC activation (Chen et al. 2010). Therefore, by affecting the CYFIP2_iso1_/WAVE1 interface, the mutation can activate the WRC aberrantly (Schaks et al. 2018; Schaks et al. 2020), matching what observed in sub‐cellular experiments (Schaks et al. 2020).

**C2**_**iso1,R87L/R87P**_ **·WRC:** L87(CYFIP2_iso1_) and P87(CYFIP2_iso1_) form weak hydrophobic interactions with Y2557(WAVE1) (Figure 5d,e). The mutations remove a hotspot. Consistently, these mutations decrease the calculated (Figure 5a) and experiment binding affinities (Nakashima et al. 2018).

**C2**_**iso1,I644M**_ **·WRC:** M664(CYFIP2_iso1_) in the mutant and I664(CYFIP2_iso1_) in the WT complex form hydrophobic interactions with W2567(WAVE1), a hotspot (Figure 3): The latter interactions are stronger than the first (Figure 5f,g). Consistently, the affinity index decreases upon mutation slightly (Figure 5a). This variant, like C2_iso1,R87C_·WRC, activates WRC aberrantly (Schaks et al. 2020). Similar arguments apply to the E665K, D724H, and Q725R variants (see below).

**C2**_**iso1,E665K**_ **·WRC:** K665(CYFIP2_iso1_) does not interact with WAVE1 (Figure 5h). In contrast, E665(CYFIP2_iso1_) forms a salt bridge with K2570(WAVE1) (Figure 5i) in the WT complex. Consistently, the affinity index decreases upon mutation (Figure 5a), supporting the hypothesis that this mutation could activate WRC aberrantly in the sub‐cellular experiment (Schaks et al. 2020). In contrast with the other mutation sites in CYFIP2_iso1_, E665(CYFIP2_iso1_) is neither a hotspot nor close to it (Figure 3).

**C2**_**iso1,D724H**_ **·WRC:** H724(CYFIP2_iso1_) forms hydrogen bonds with Q2516(WAVE1) and D2656(WAVE1) (Figure 5j) and disrupts the H‐bond between R795(CYFIP2_iso1_) (a hotspot) and Q2515(WAVE1) (Figures 3 and 5j,k). In the WT complex, hotspot residue D724(CYFIP2_iso1_) forms H‐bonds with Q2515(WAVE1) and Q2516(WAVE1) (Figures 3 and 5k). The affinity index decreases (Figure 5a) consistently with removing a hotspot and with the experimentally observed aberrant C2_iso1,D724H_·WRC activation (Schaks et al. 2020).

**C2**_**iso1,Q725R**_ **·WRC:** R725(CYFIP2_iso1_) does not interact with WAVE1 (Figure 5l). In the WT complex, instead, Q725 forms a hydrogen bond with W2567(WAVE1) (Figure 5m), a hotspot (Figure 3). Thus, the disruption of the interactions with the latter, a hotspot, might significantly affect the binding, as observed in the decrease of the affinity index (Figure 5a). This supports the findings that this mutation aberrantly activates WRC in the subcellular experiments (Schaks et al. 2020).

##### CYFIP2_iso1_ /NCKAP1_iso1_ interface

**FIGURE 6:**
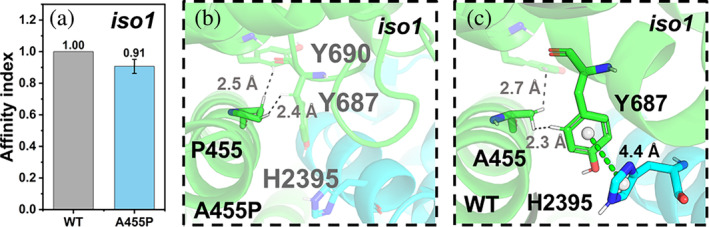
A455P variant at the C2_iso1_·WRC complex. (a) Affinity indexes. The values are represented as means ± SD (*N* = 3). (b, c) Alterations associated with the mutant. For the mutant, the color scheme as in Figure 5. In addition, the π–π interaction is shown in green dashed lines.

**C2**_**iso1,A455P**_ **·WRC:** The residue at position 455 is not located at the interface, but it is close to it. The mutation turns out to break H2395(NCKAP1_iso1_)/Y687(CYFIP2_iso1_) π–π stacking interactions (Figure 6b,c). The affinity index decreases (Figure 6a) in accordance with the fact that Y687 is a hotspot (Figure 3). The mutation can activate the WRC through potential allosteric regulation (Schaks et al. 2020). Our predicted models offer structural insights into this potential mechanism: the A455P mutation could impair the binding between CYFIP2_iso1_ and NCKAP1_iso1_ indirectly by weakening the interactions of the hotspot, possibly resulting in the disruption of CYFIP2_iso1_/WAVE1 interactions and consequent unwanted activation of the WRC (Schaks et al. 2020).

##### CYFIP2_iso2_ /WAVE1 interface

**FIGURE 7:**
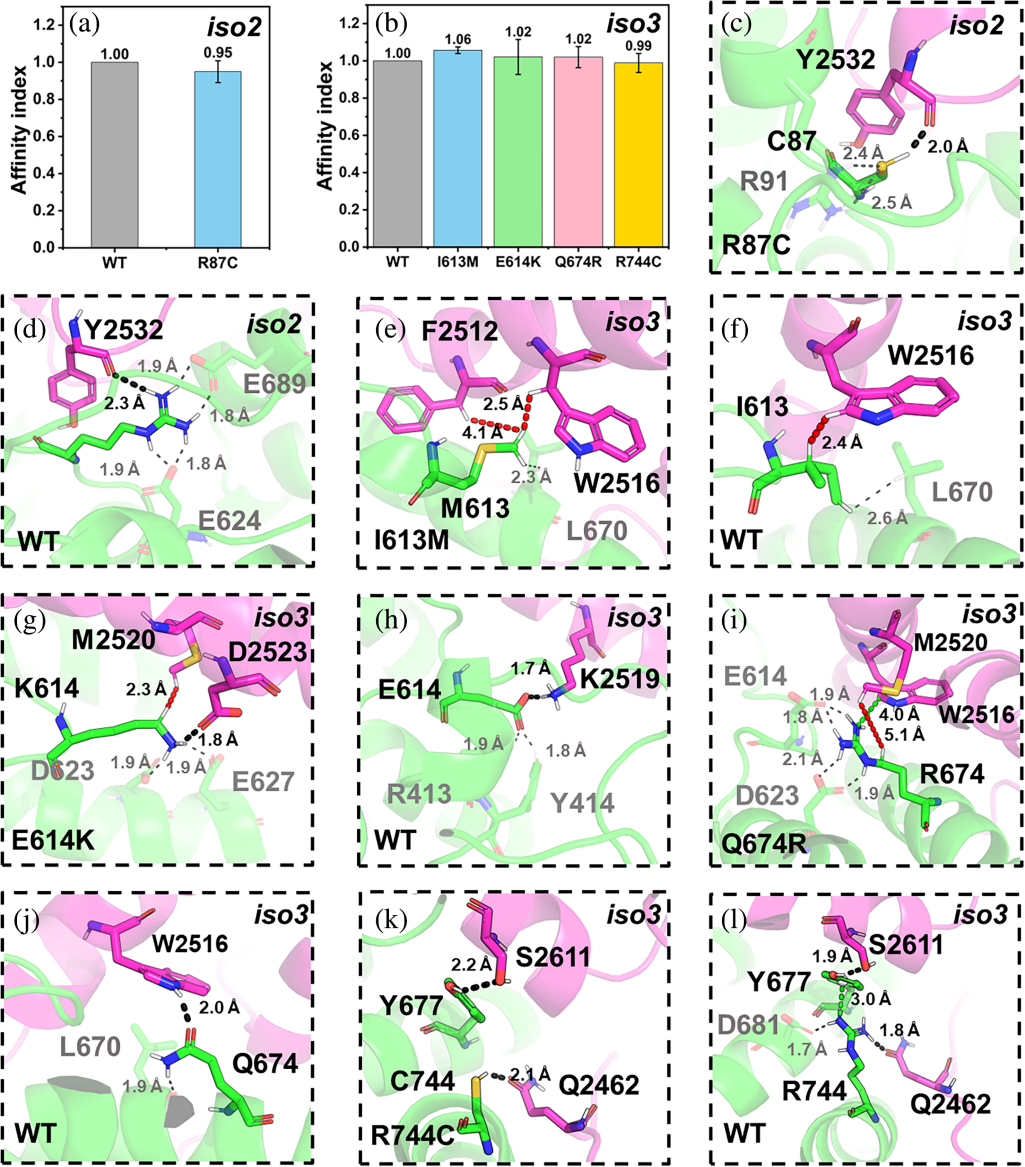
ASD‐related mutations at the CYFIP2_iso2–3_/WAVE1 interface. (a, b) The affinity indexes for the WT and the mutants at the interface. The values are represented as mean ± SD (*N* = 3). (c–l) Alterations associated with R87C(CYFIP2_iso2_) (c, d), I613M(CYFIP2_iso3_) (e, f), E614K(CYFIP2_iso3_) (g, h), Q674R(CYFIP2_iso3_) (i, j) R744C(CYFIP2_iso3_) (k, l). The color scheme as in Figure 5. Cation–π interactions are shown as a green dashed line.

**C2**_**iso2,R87C**_ **·WRC:** The interaction patterns for both the mutant and WT (Figure 7c,d) complexes are highly similar to those of WT and R87C C2_iso1_·WRC (Figure 5b,c). The mutation removes the hotspot R87 (Figure S5a), and accordingly, the affinity index also decreases (Figure 7a). Therefore, this mutation should have similar results to the C2_iso1,R87C_·WRC variant. Our results are consistent with previous molecular dynamics (MD) simulations carried out on CYFIP2_iso2_/NCKAP1_iso1_/WAVE1 complex (thus lacking HSPC300 and ABI2 proteins in Figure 2), which also suggest that R87C mutations destabilize CFYIP2_iso2_/WAVE1 interactions (Biembengut et al. 2022).

##### CYFIP2_iso3_ /WAVE1 interface

**C2**_**iso3,I613M**_ **·WRC:** For this and the two mutants below, the interaction pattern differs from that of C2_iso1,WT_·WRC. Here, M613(CYFIP2_iso3_) forms hydrophobic interactions with F2512(WAVE1) and W2516(WAVE1) (Figure 7e), two hotspots (Figure S5b), while in the WT I613(CYFIP2_iso3_) interacts only with W2516(WAVE1) (Figure 7f). Consistently, the affinity index increased (Figure 7b).

**C2**_**iso3,E614K**_ **·WRC:** K614(CYFIP2_iso3_) forms a salt bridge with D2523(WAVE1) and van der Waals interactions with M2520(WAVE1) (Figure 7g). In isoform 1, the correspondent residue (K665) does not interact with any WAVE1 residue (Figure 5h). In the WT complex, E614(CYFIP2_iso3_) is not a hotspot, and there are no other hotspots within 4 Å. It forms only a salt bridge with K2519(WAVE1) (Figure 7h). The affinity index increases slightly (Figure 7b).

**C2**_**iso3,Q674R**_ **·WRC:** R674(CYFIP2_iso3_) forms a cation–π interaction with W2516(WAVE1) (Figure 7i), a hotspot, and van der Waals interactions with M2520(WAVE1).^2^ Q674(CYFIP2_iso3_), albeit a hotspot (Figure S5b), forms only a hydrogen bond with W2516(WAVE1) (Figure 7j). Consistently, the affinity index increases upon mutation, albeit only slightly (Figure 7b).

The observed structural and affinity index (Figures 5 and 7) differences between the above three variants and their corresponding mutants in CYFIP2_iso1_ may be attributed, at least in part, to the presence of the MR2 region. The latter interacts with the α4‐ and α5‐helices immediately adjacent to the α6‐ and C‐helices of WAVE1. The α6‐ and C‐helices are the interacting regions for these mutations aforementioned in this section (Figure 2).

The increased binding between WCA and CYFIP2 upon mutations I613M(CYFIP2_iso3_), E614K(CYFIP2_iso3_), Q674R(CYFIP2_iso3_) might also affect WRC activation, though no direct experimental evidence is currently available.

**C2**_**iso3,R744C**_ **·WRC:** C744(CYFIP2_iso3_) forms a hydrogen bond with Q2462(WAVE1) (Figure 7k). In the WT complex, R744(CYFIP2_iso3_) forms a stronger hydrogen bond with the same residue (Q2462(WAVE1)) (Figure 7l) and a cation–π interaction with Y677(CYFIP2_iso3_), which is a hotspot (Figure S5b). In addition, the R744C mutation weakens the hydrogen bond between Y677(CYFIP2_iso3_) and S2611(WAVE1). Accordingly, the affinity index decreased, albeit slightly (Figure 7b).

##### CYFIP2_iso3_ /NCAKP1_iso1_ interface

**FIGURE 8:**
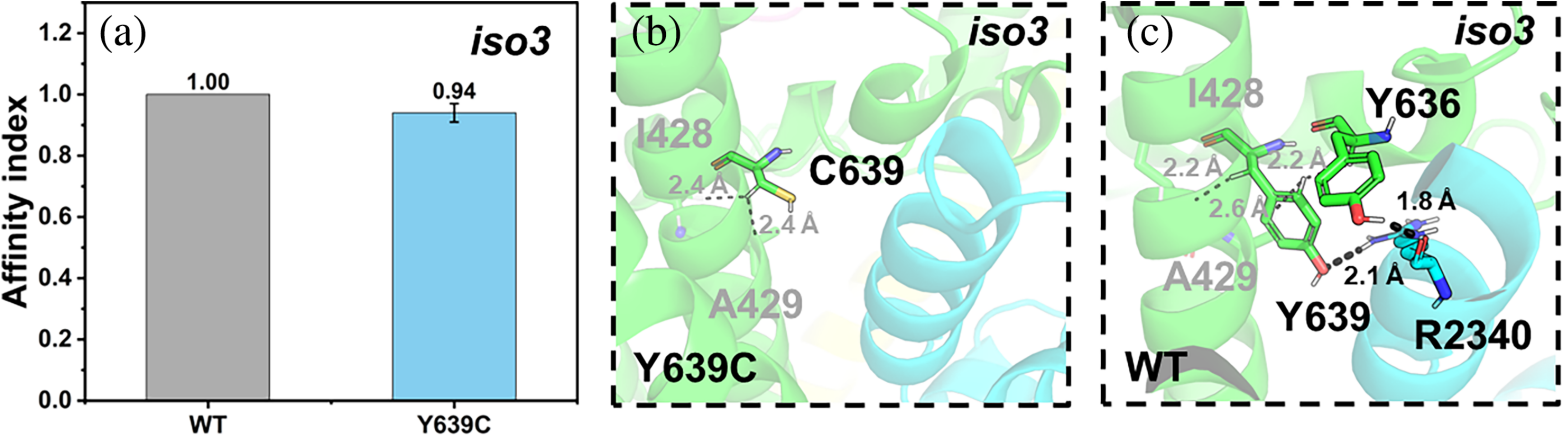
Y639 variant at the C2_iso3_·WRC complex. (a) Affinity indexes. The values are represented as mean ± SD (*N* = 3)). (b, c) Alterations associated with the mutations. The color scheme as in Figure 5.

**C2**_**iso3,Y639C**_ **·WRC:** C639(CYFIP2_iso3_) does not interact with NCKAP1_iso1_ (Figure 8b). In the WT complex, Y639(CYFIP2_iso3_) forms a hydrogen bond with a hotspot of NCKAP1_iso1_, R2340 (Figures 8c and S5b). Consistently, the affinity index decreases (Figure 8a).

### 2.2 N1_iso2_·WRC variants

We constructed the N1_iso1,WT_·WRC and N1_iso2,WT_·WRC complexes, starting from the same X‐ray structure as above (PDB ID: 3P8C (Chen et al. 2010)), and following the same protocol (Table S8). As expected, also here the CYFIP2/NCKAP1 and CYFIP2/WAVE1 contact surfaces feature the largest number of hotspots (by far) among the eight protein/protein surfaces of WRC (Figure S14). We constructed the mutants in Table 1 following the same scheme as above.

The only difference between N1_iso1,WT_·WRC and N1_iso2,WT_·WRC is an additional loop (^36^KQGQVW^41^) in the latter, which is far away from the CYFIP2/NCKAP1 interface involved in the 2 NCKAP1_iso2_ mutations studied here (Table 1 and Figure 2c). As expected, the contact maps are similar to those of C2_iso1–3,WT_·WRC (Figures S15–S17).

**FIGURE 9:**
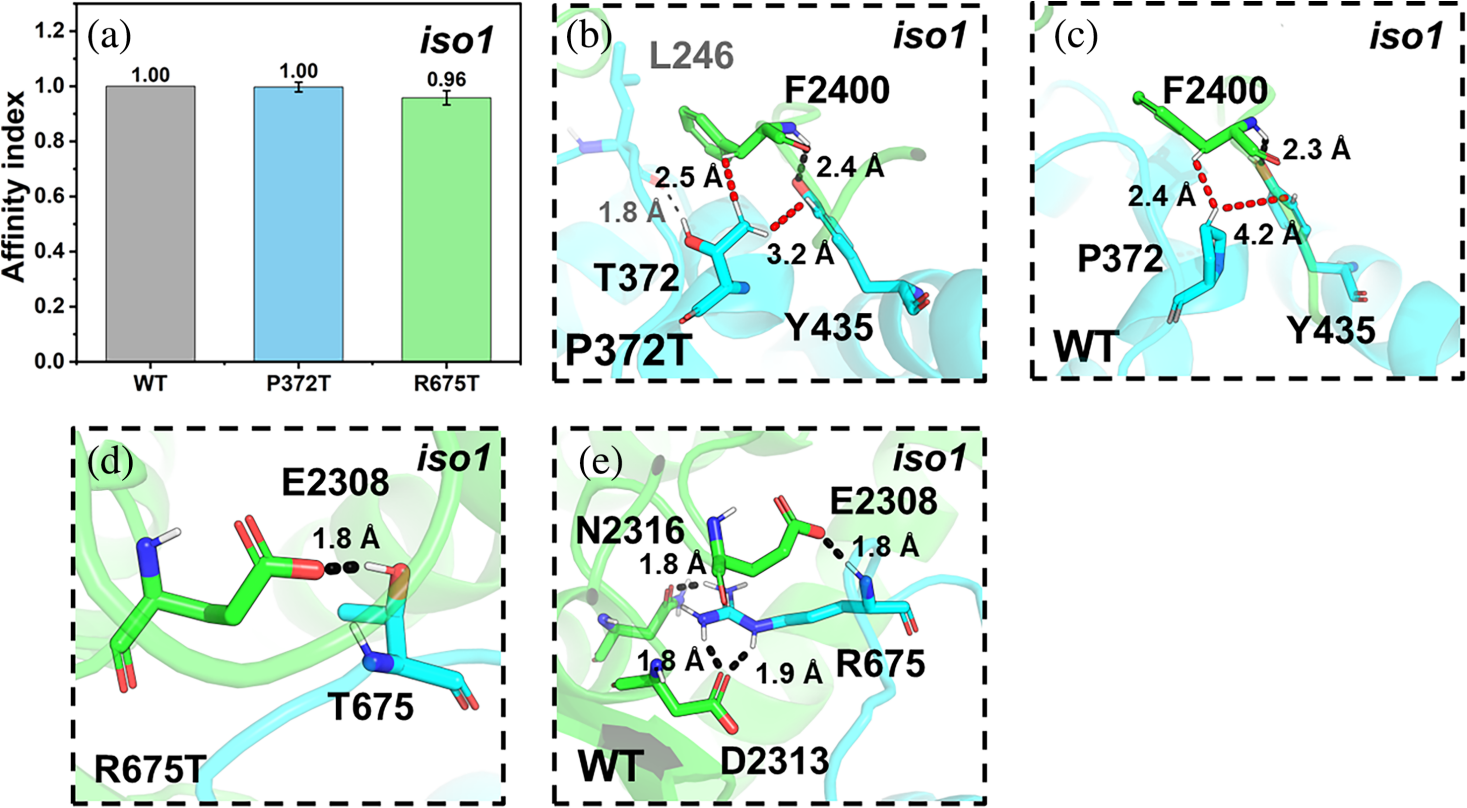
ASD‐related mutations at the CYFIP2_iso1_/NCKAP1_iso2_ interface. (a) The affinity indexes of the mutants. Values are represented as mean ± SD (*N* = 3). (b–e) Alterations associated with the P372T (b, c) and R675T (d, e) mutations.

**N1**_**iso2,P372T**_ **·WRC:** T372(NCKAP1_iso2_) forms hydrophobic interactions with F2400(CYFIP2_iso1_), a hotspot at the CYFIP2_iso1_/NCKAP1_iso2_ interface (Figure 9b). In the WT complex, P372(NCKAP1_iso2_) forms hydrophobic interactions with the same residue (F2400(CYFIP2_iso1_)) (Figure 9c). The affinity index does not change (Figure 9a).

**N1**_**iso2,R675T**_ **·WRC:** T675(NCKAP1_iso2_) forms a hydrogen bond with E2308(CYFIP2_iso1_) (Figure 9d). In the WT complex (Figure 9e), R675(NCKAP1_iso2_) forms a salt bridge with E2308(CYFIP2_iso1_), one with D2313(CYFIP2_iso1_), and a hydrogen bond with N2316(CYFIP2_iso1_). R675(NCKAP1_iso2_) is a hotspot (Figure S14a); hence, its replacement with threonine may affect the interface significantly. Consistently, the affinity decreases (Figure 9a).

As expected, the correspondent results for the N1_iso2_·WRC variants with the other two isoforms of CYFIP2 are very similar (Figures S18 and S19).

## 3 CONCLUSIONS

Our calculations show that the mutations in Table 1 are located in the protein/protein interfaces tightly bound to each other by the largest number of hotspots. For the first time, we mapped these mutations on the correct isoforms of CYFIP2 and NCKAP1, which is critical given the different distribution of the isoforms in human tissues (Patowary et al. 2024). The mutants affect the stability of the CYFIP2/WAVE1 or /NCKAP1 interface by removing hotspots (R87C/L/P(CYFIP2_iso1–2_), D724H(CYFIP2_iso1_), and Q674R(CYFIP2_iso3_)) or indirectly (A455P(CYFIP2_iso1_), Y639C(CYFIP2_iso3_), I613M(CYFIP2_iso3_), I664M(CYFIP2_iso1_), Q725R(CYFIP2_iso1_), R744C(CYFIP2_iso3_), R675T(NCKAP1_iso2_)), by affecting the interaction networks of other hotspots. Furthermore, our work provides insight into their effect at the molecular level. Our approach, albeit approximate, goes beyond simple homology modeling, and it is fully consistent with the experiment: in particular, we predict that most mutations in Table 1 weaken the interactions of CYFIP2_iso1–3_ with their cellular partners WAVE1 and NCKAP1_iso1–2_. Specifically, R87C(CYFIP2_iso1–2_), R87L(CYFIP2_iso1_), R87P(CYFIP2_iso1_), I664M(CYFIP2_iso1_), E665K(CYFIP2_iso1_), D724H(CYFIP2_iso1_), Q725R(CYFIP2_iso1_), and R744C(CYFIP2_iso3_), affect CYFIP2/WAVE1 binding, while A455P(CYFIP2_iso1_), Y639C(CYFIP2_iso3_), and R675T(NCKAP1_iso2_) mutations affect the CYFIP2/NCKAP1 interface. These results provide molecular insights for rationalizing subcellular experiments, which demonstrated that R87C(CYFIP2_iso1_), A455P(CYFIP2_iso1_), I664M(CYFIP2_iso1_), E665K(CYFIP2_iso1_), D724H(CYFIP2_iso1_), and Q725R(CYFIP2_iso1_) variants cause abnormal WRC activation (Schaks et al. 2020). We predict here that R87C(CYFIP2_iso2_), R87L(CYFIP2_iso1_), R87P(CYFIP2_iso1_), Y639C(CYFIP2_iso3_), R744C(CYFIP2_iso3_), and R675T(NCKAP1_iso2_) could also activate WRC aberrantly. Interestingly, a few mutations turn out to strengthen the interactions between the CYFIP2_iso3_ and WAVE1 (Table 1). We assume that this reinforcement of the interactions may also impair WRC activation by making the release of the WCA region more difficult, potentially affecting downstream actin remodeling. Interestingly, the effect of mutations on the strength of the interaction changes, at times, when passing from CYFIP2_iso3_ to CYFIP2_iso1_ (Table 1 and Figures 5 and 7): according to our data, the mutations I613M, E614K, and Q674R in CYFIP2_iso3_ strengthen the interaction networks of the CYFIP2/WAVE1 interface while the corresponding mutations in CYFIP2_iso1_ (E664M, E665K, and Q725R) weaken them. Moreover, CYFIP2_iso1_ exhibits a higher predicted affinity for WAVE1 than the other two isoforms (Figures S20 and S21; see Data S1 for details). Thus, the three isoforms of CYFIP2 might exhibit different efficiencies in the WRC pathway (Figure S1). Our results suggest that it is key to consider the correct isoforms of the proteins in terms of the formation of complexes and the impact of disease‐related mutations.

## 4 METHODS

### 4.1 CYFIP2_iso1–3_ variants

These were predicted for the CYFIP2_iso1–3_ and NCKAP1_iso1_ in the WRC (C2_iso1–3_·WRC hereafter). We followed a multistep strategy. First, we predicted the structural determinants of the WT complex based on the X‐ray structure at 2.3 Å resolution of the structurally similar CYFIP1_iso1_ protein (sequence identity as high as 88%, Table S4) in the WRC (PDB ID: 3P8C) (Chen et al. 2010). The SWISS‐MODEL (Waterhouse et al. 2018) and MODELLER V9.19 (Webb and Sali 2021) codes were used. Notice that we do not know which isoforms are involved in the ASD‐linked variants of WRC at present. Thus, we used the canonical isoforms of ABI2, HSPC300, WAVE1, and NCKAP1 in the 3P8C structure (Table S9). Next, we established a protocol for building the variants using docking procedures between WT CYFIP2_iso1–3_ and the rest of the complex (Figure 4).

- **Blind docking of CYFIP2**_**iso1–3**_ **onto the rest of the complex** was performed by the GRAMM web server (Singh et al. 2024). The number of scans that match the output was set to 300,000, and the top 10 predicted structures were considered for successive refinement.
- **Refinement of the 10 structures** by reducing atomic clashes and optimizing binding poses, followed by minimization using the relax function with InterfaceRelax 2019 parameters. This step was performed with Rosetta 2021.16 (Khatib et al. 2011; Maguire et al. 2021; Tyka et al. 2011).
- **Energy‐based re‐ranking** based on Molecular Mechanics‐Generalized Born Surface Area (MM‐GBSA) (Wang et al. 2019) via the sander and pmemd engines of AMBER22 software (Case et al. 2022). The ff02 force field (Cieplak et al. 2001) and the GB^OBC1^ model (Onufriev et al. 2004) were used to predict protein–protein affinity, as previous benchmark studies demonstrated they have the best performance in predicting protein–protein complexes (Chen et al. 2016).

In this protocol, we use MM‐GBSA calculations to evaluate the affinity of the protein–protein interactions in the C2_iso1–3,WT_·WRC and N1_iso2,WT_·WRC and their ASD‐linked variants. In particular, we use the enthalpy component calculated in this approach as affinity index (which is here taken in its absolute value and normalized to the WT), Δ*H*_MM‐GBSA_ the value 1.0 refers to the best‐docked conformation of the WT complexes regarding Δ*H*_MM‐GBSA_. Besides being very approximate, Δ*H*_MM‐GBSA_ does not include entropy. Assuming that this contribution does not vary mainly on passing from one complex to the other, we then use this quantity as a qualitative index of stability of protein/protein interface: namely, we use Δ*H*_MM‐GBSA_ values to establish qualitatively the stability of the mutated complexes relative to the WT structures.

Our protocol was first validated with the WT complexes. It reproduced the structural determinants of the WT WRC, which have been built as described in the previous section (see Data S1 for details). Then, we applied the protocol to the ASD‐linked variants of the protein (Table S10). Only residues at the interface in WT WRC were considered, except for A455(CYFIP2_iso1_), as this causes aberrant lamellipodia, which may have an apparent effect on the stabilization at the interface (Table 1).

The CPPTRAJ (Roe and Cheatham III 2013), a module of AMBER software, was used to calculate the Cα‐atom root‐mean‐square deviation (RMSD) values and contact map. The interface residues are listed in Tables S5–S7 and S11–S13. The residues within 4 Å are considered as interacting or close to each other. The hydrogen bonding interactions are defined as if the distance between the donor atom (D) and the acceptor atom (A) is less than 3.5 Å and the D‐H‐A angle is larger than 135°. The cation–π interactions are identified by the following criterion: the distance between the cation and the center of the centroid of the π‐system is 5 Å or lower.

### 4.2 NCKAP1_iso2_ variants

The ASD‐linked mutations at the CYFIP2/NCKAP1 interface are only mapped on NCKAP1_iso2_. NCKAP1_iso2_ variants were predicted for the complex of this protein with CYFIP2_iso1–3_, along with the other proteins in the WRC (N1_iso2_·WRC hereafter). We followed the same multistep strategy as above, except that in the homology modeling, we also predicted the structural determinants of NCKAP1_iso2_, which is very similar to NCKAP1_iso1_ (99.5% identity), being the only difference K36 replaced by the ^36^KQGQVWK^42^ loop. In addition, we validated the procedure to generate the WT complex again using NCKAP1_iso2_.

### 4.3 Hotspot predictions

These were performed on the C2_iso1–3,WT_·WRC and N1_iso2,WT_·WRC complexes using the mCSM (Pires et al. 2013), BeAtMuSiC (Dehouck et al. 2013), and FoldX (Schymkowitz et al. 2005) servers. The hotspots are defined as the interface residues whose substitution with an alanine leads to a loss of protein/protein binding free energy of 2 kcal/mol or larger (Cukuroglu et al. 2014).

## AUTHOR CONTRIBUTIONS

**Song Xie:** Methodology; writing – original draft; writing – review and editing; visualization; formal analysis. **Ke Zuo:** Formal analysis; writing – original draft; writing – review and editing. **Silvia De Rubeis:** Conceptualization; writing – review and editing. **Paolo Ruggerone:** Conceptualization; funding acquisition; supervision; formal analysis; writing – review and editing. **Paolo Carloni:** Conceptualization; writing – original draft; writing – review and editing; investigation; funding acquisition.

## Supporting information


**Data S1.** Supporting Information.

## Footnotes

1 As mentioned above, mutations of WAVE1, HSPC300, and ABI2 proteins have no impact on ASD (Fu et al. 2022). Thus, here we considered only their WT canonical isoforms.

2 In isoform 1 (Figure 5l), the residues corresponding to R674 (R725) do not interact with WAVE1.
