# Supplementary material for: Molecular basis of the CYFIP2 and NCKAP1 autism‐linked variants in the WAVE regulatory complex
Source: Protein Sci. 2024 Dec 11;34(1):e5238. doi: 10.1002/pro.5238 (PMC11632847; doi:10.1002/pro.5238)
Supplement: Supplementary file 1 — Data S1. Supporting Information. [file PRO-34-e5238-s001.docx]

**SUPPLEMENTARY INFORMATION**

**Molecular basis of the CYFIP2 and NCKAP1 autism-linked variants in the WAVE regulatory complex**

Song Xie^1, 2^, Ke Zuo^1, 3, 4, *^, Silvia De Rubeis^5, 6, 7, 8^, Paolo Ruggerone^4, *^, Paolo Carloni^1, 2, 9 *^

1 Computational Biomedicine, Institute of Advanced Simulation IAS‑5 and Institute of Neuroscience and Medicine INM‑9, Forschungszentrum Jülich GmbH, Jülich, 52428, Germany.

2 Department of Physics, RWTH Aachen University, Aachen, 52074, Germany.

3 National & Local Joint Engineering Research Center of Targeted and Innovative Therapeutics, Chongqing Key Laboratory of Kinase Modulators as Innovative Medicine, College of Pharmacy (International Academy of Targeted Therapeutics and Innovation), Chongqing University of Arts and Sciences, Chongqing, 402160, PR China.

4 Department of Physics, University of Cagliari, Citt. Universitaria, I-09042 Monserrato (Cagliari), Italy.

5 Seaver Autism Center for Research and Treatment, Icahn School of Medicine at Mount Sinai, 10029-5674, New York, USA.

6 Department of Psychiatry, Icahn School of Medicine at Mount Sinai, 10029-5674, New York, USA.

7 The Mindich Child Health and Development Institute, 10029-5674, Icahn School of Medicine at Mount Sinai, New York, USA.

8 Friedman Brain Institute, Icahn School of Medicine at Mount Sinai, 10029-5674, New York, USA.

9 JARA Institute: Molecular Neuroscience and Imaging, Institute of Neuroscience and Medicine INM‑11, Forschungszentrum Jülich GmbH, 52428, Jülich, Germany.

*Corresponding author. E-mail: [k.zuo@stimulate-ejd.eu](mailto:k.zuo@stimulate-ejd.eu) (Ke Zuo); paolo.ruggerone@dsf.unica.it (Paolo Ruggerone); p.carloni@fz-juelich.de (Paolo Carloni)

**1. Additional information on the protocol validation.**

*1.1 Details on the model refinement.* Clashes between residues of the CYFIP2_iso1_ and NCKAP1_iso1_/WAVE1/HSPC300/ABI2 surfaces were identified in the top ten-ranked docking models obtained using the GRAMM webserver (Singh *et al.* 2024). They were removed by refining the models using Rosetta. The ResProx values (Berjanskii *et al.* 2012) turned out to be rather small (**Fig. S8A**), pointing to the high quality of the models. The RMSD values between Cα atoms of the C2_iso1_·WRC models and the corresponding homology models ranged between 1.4 Å and 1.9 Å (**Fig. S8B**).

*1.2 Details on the structural predictions of C2_iso1-3, WT_·WRC and N1_iso2, WT_·WRC.* The Cα RMSD values among them ranged from 1.3 Å to 1.7 Å (**Tab. S10**) and 2.1 to 2.5 Å (**Tab. S7**), respectively, suggesting overall similar structural determinants and validating the robustness of our protocol.

*1.3 Structural Prediction of the p.Arg87Cys C2_iso1_·WRC variant using two different approaches.* Here, we used our protocol and the standard SWISS-MODEL (Waterhouse *et al.* 2018)/Modeller V9.19 (Webb and Sali 2021) procedure based on X-ray structure (PDB ID: 3P8C (Chen *et al.* 2010))**.** We find that the interaction between C87 and Y2557 in WAVE1 (**Fig. S9**) occurs only in our procedure.

**2. Binding strength of CYFIP2_iso1-3_ for WAVE1**.

The three different CYFIP2 isoforms may have different efficacies in activating WRC, as (i) the efficacy may depend on the strength of their interactions with other interactors in WRC, such as WAVE1 and (ii) CYFIP2_iso1_ exhibits the largest affinity index for WAVE1, followed by CYFIP2_iso2_ and, finally, by CYFIP2_iso3_ (**Fig. S20**). These differences might be caused, at least in part, by the MR1(CYFIP2_iso1_/WAVE1) interactions (**Fig. S21**), absent in the other two isoforms and by the MR2(CYFIP2_iso1-2_/WAVE1) interactions, absent in the isoform 3.

**3. Supplementary Figures**

**
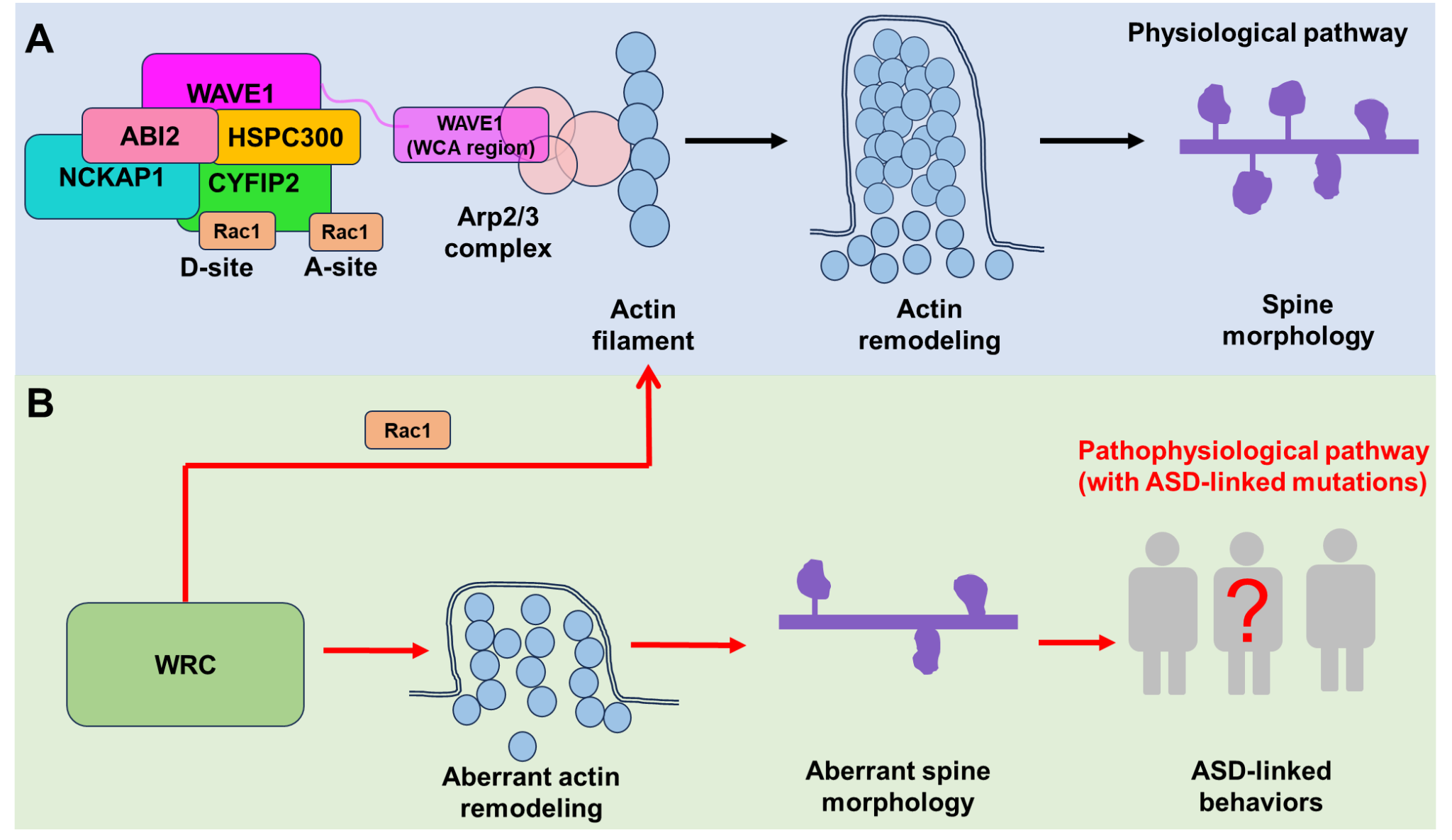
**

**Fig. S1. (A) The WRC-mediated pathway to actin remodeling. (B**) **In the case of specific CYFIP2 variants (Tab. 1), this pathway can lead to ASD.** (**A)** Rac1 binding to CYFIP2 regulates the release of the VCA region of the WAVE1 protein (WCA). For part of the complex, cryo-EM structural information is available for a structurally similar complex (Ding *et al.* 2022), while the rest (in lighter colors) is based on biological information (Takenawa and Suetsugu 2007; Chen *et al.* 2010; Schaks *et al.* 2020). The complex binds to the Arp2/3 complex, which modulates the actin filament (Takenawa and Suetsugu 2007; Chen *et al.* 2010). Binding of another activator (Arf1) (Yang *et al.* 2022) to WRC can also modulate actin remodelling. (**B**) The specific ASD-linked variants of WRC may release the WCA before Rac1 binding (Schaks *et al.* 2020). If Rac1 does not bind to the WRC complexes, aberrant lamellipodia (Schaks *et al.* 2020) and spine morphology are formed. Eventually, this leads to ASD-linked behaviors (Bardoni and Abekhoukh 2014). The structural determinants of the variant complex are unclear.


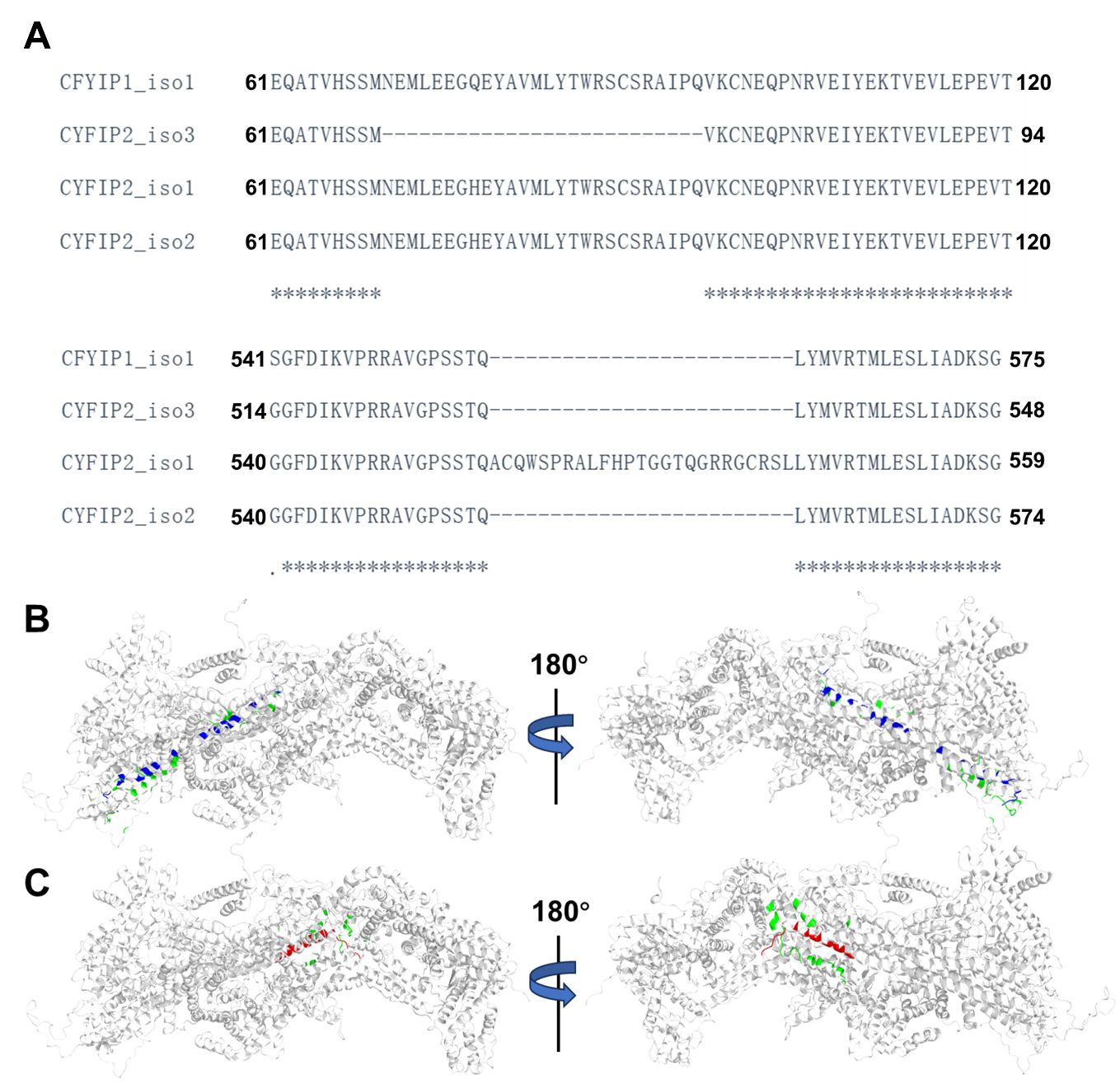


**Fig. S2. (A) The sequence difference between CYFIP1 and three CYFIP2 isoforms, cartoon representation of the (B) HSPC300/CYFIP2_iso1_ and (C) ABI2/CYFIP2_iso2_ contact surfaces**; The CYFIP2, HSPC300, and ABI2 contact surfaces are in green, blue, and red, respectively. CYFIP2_iso2_ has the largest similarity and identity with CYFIP1_iso1_ (**Table S9**). The MUSCLE (Edgar 2004) is used to perform sequence alignment.

**
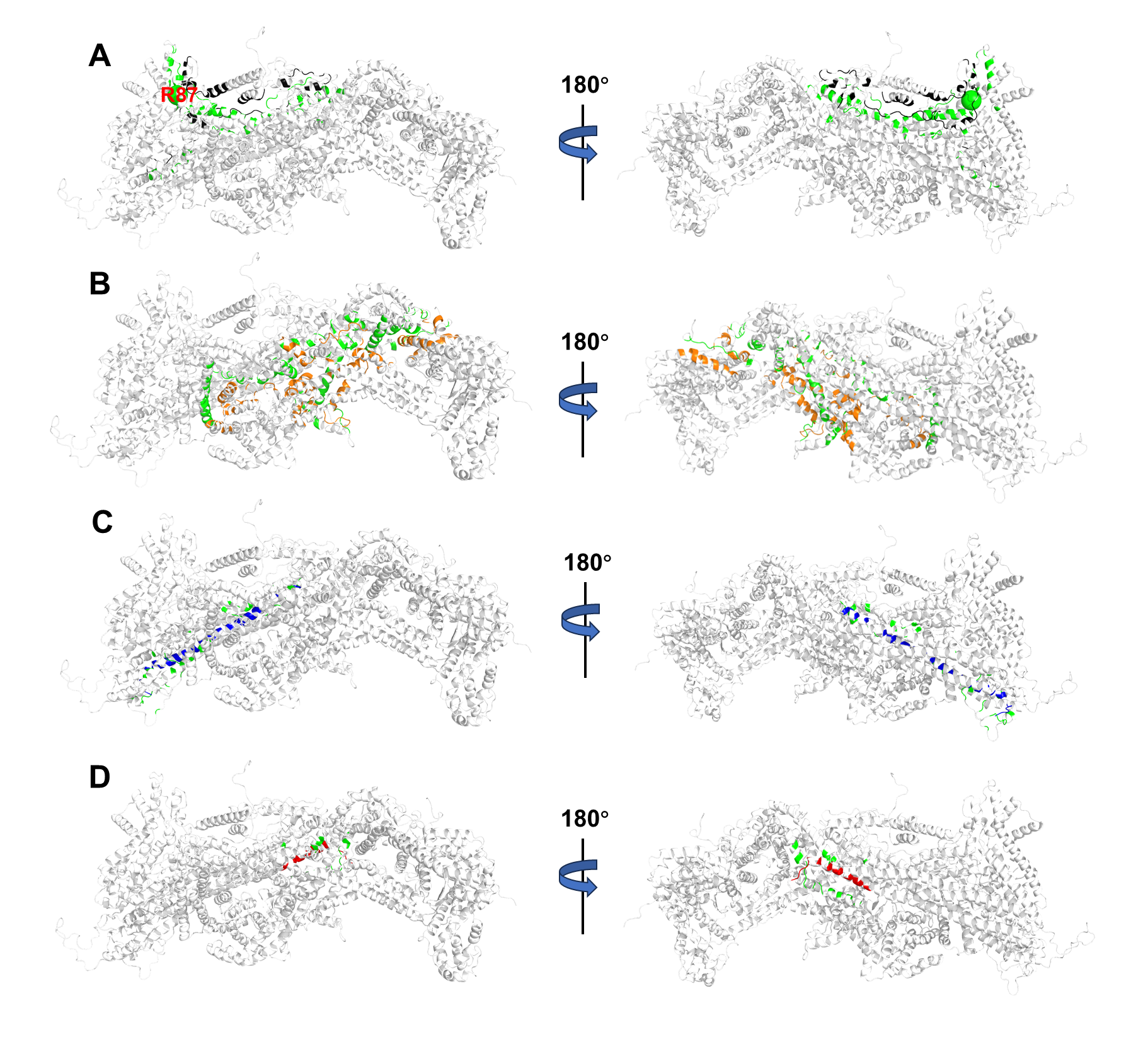
**

**Fig. S3. Structural prediction of C2_iso2, WT_·WRC.** (A) Cartoon representation of the contact surfaces of (A) WAVE1/CYFIP2_iso2_, (B) NCKAP1_iso1_/CYFIP2_iso2_, (C) HSPC300/CYFIP2_iso2_, and (D) HSPC300/CYFIP2_iso2_. The CYFIP2, WAVE1, NCKAP1_iso1_, HSPC300, and ABI2 contact surfaces are in green, black, orange, blue, and red, respectively. The Cα atom of the R87 is shown as a large sphere and labeled in panel A.


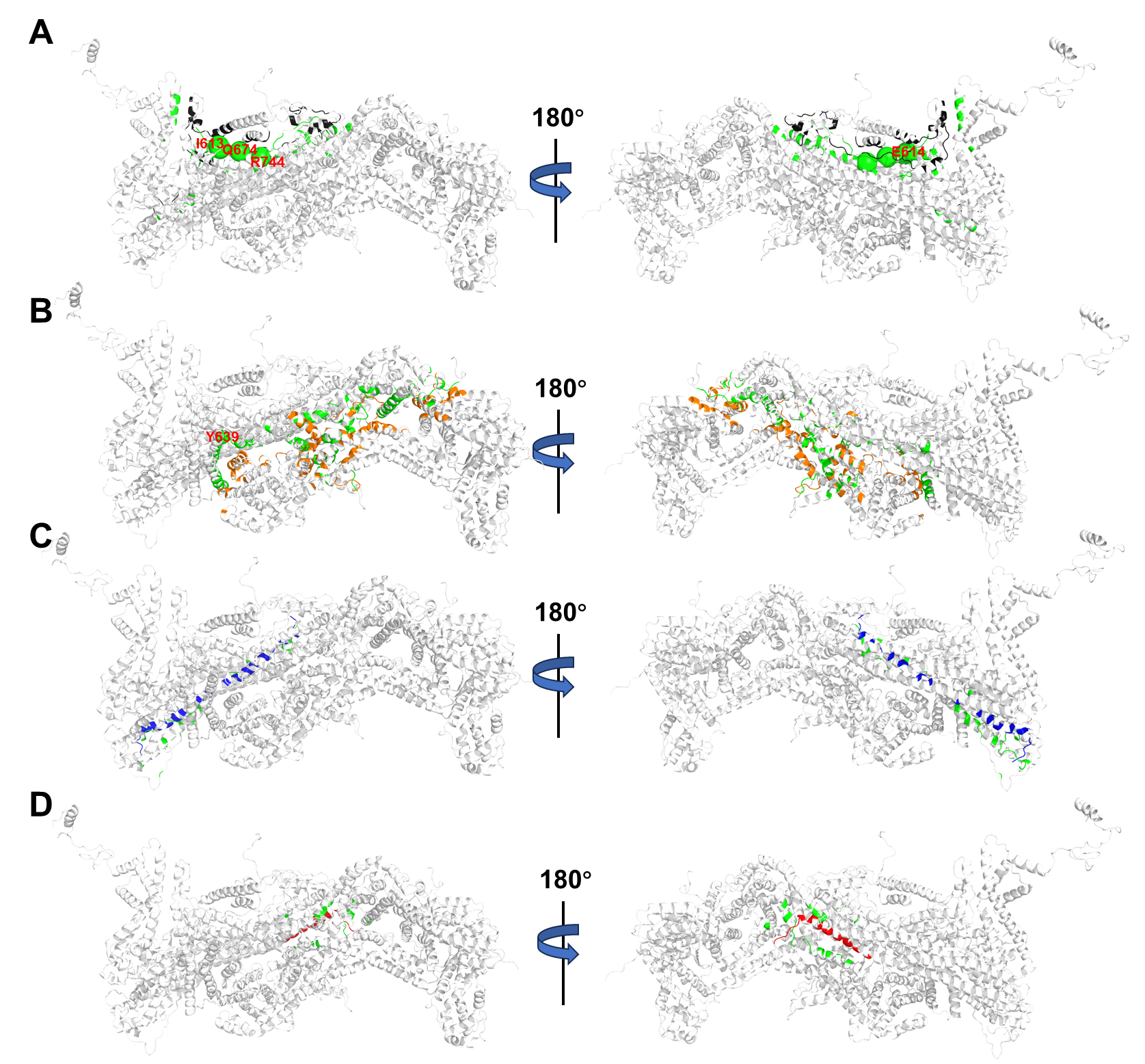


**Fig. S4. Structural prediction of C2_iso3, WT_·WRC.** (A) Cartoon representation of the contact surfaces of (A) WAVE1/CYFIP2_iso3_, (B) NCKAP1_iso1_/CYFIP2_iso3_ (C), HSPC300/CYFIP2_iso3,_ and (D) HSPC300/CYFIP2_iso3_. The color scheme and labeling I613, Q674, R744, and Y639 are the same as in **Fig. S3.**


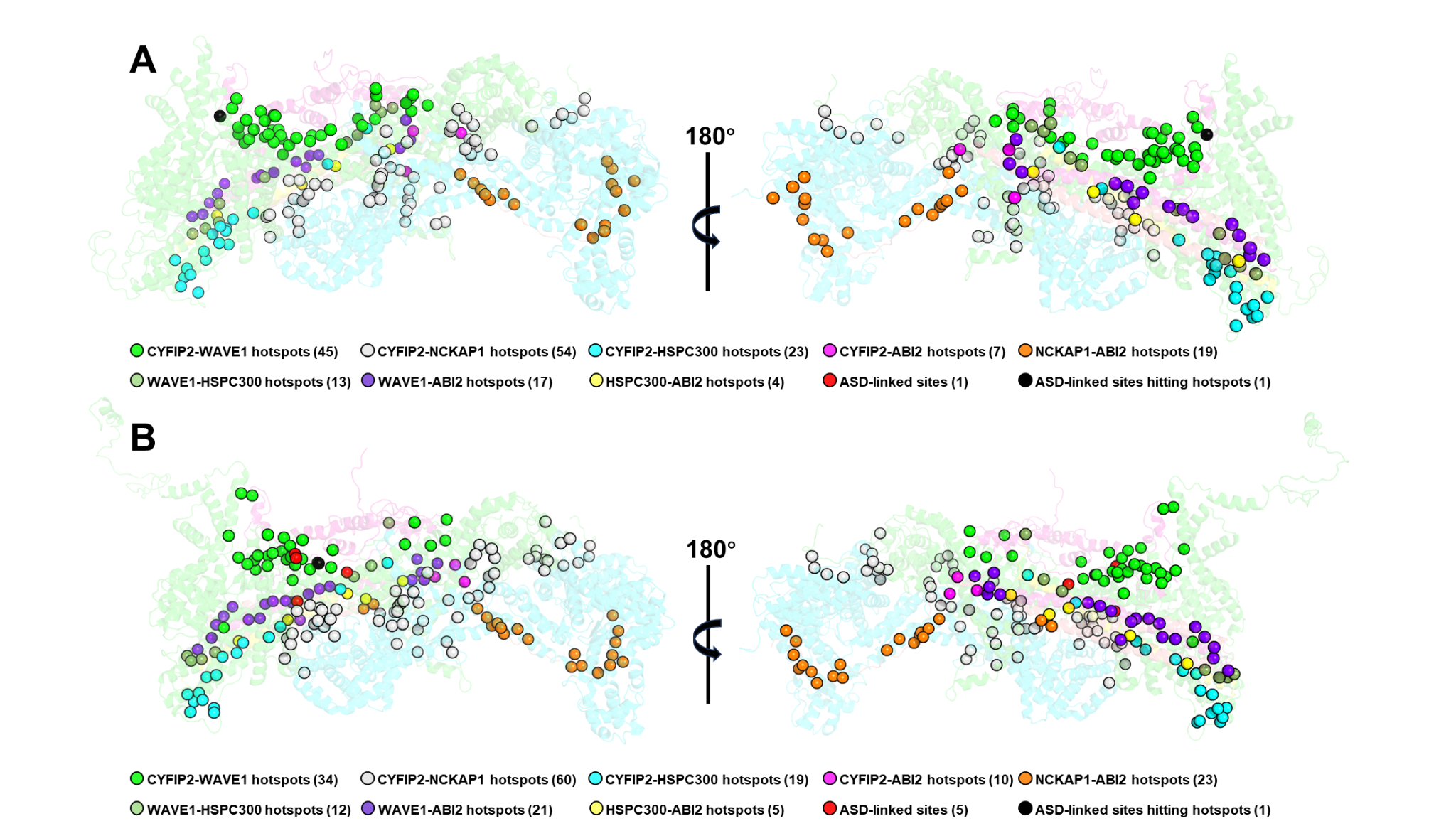


**Fig. S5. (A-B) Hotspots prediction of C2_iso2 and 3, WT_·WRC by mCSM (Pires *et al.* 2013).** The color scheme is as in **Fig. 3**.

**
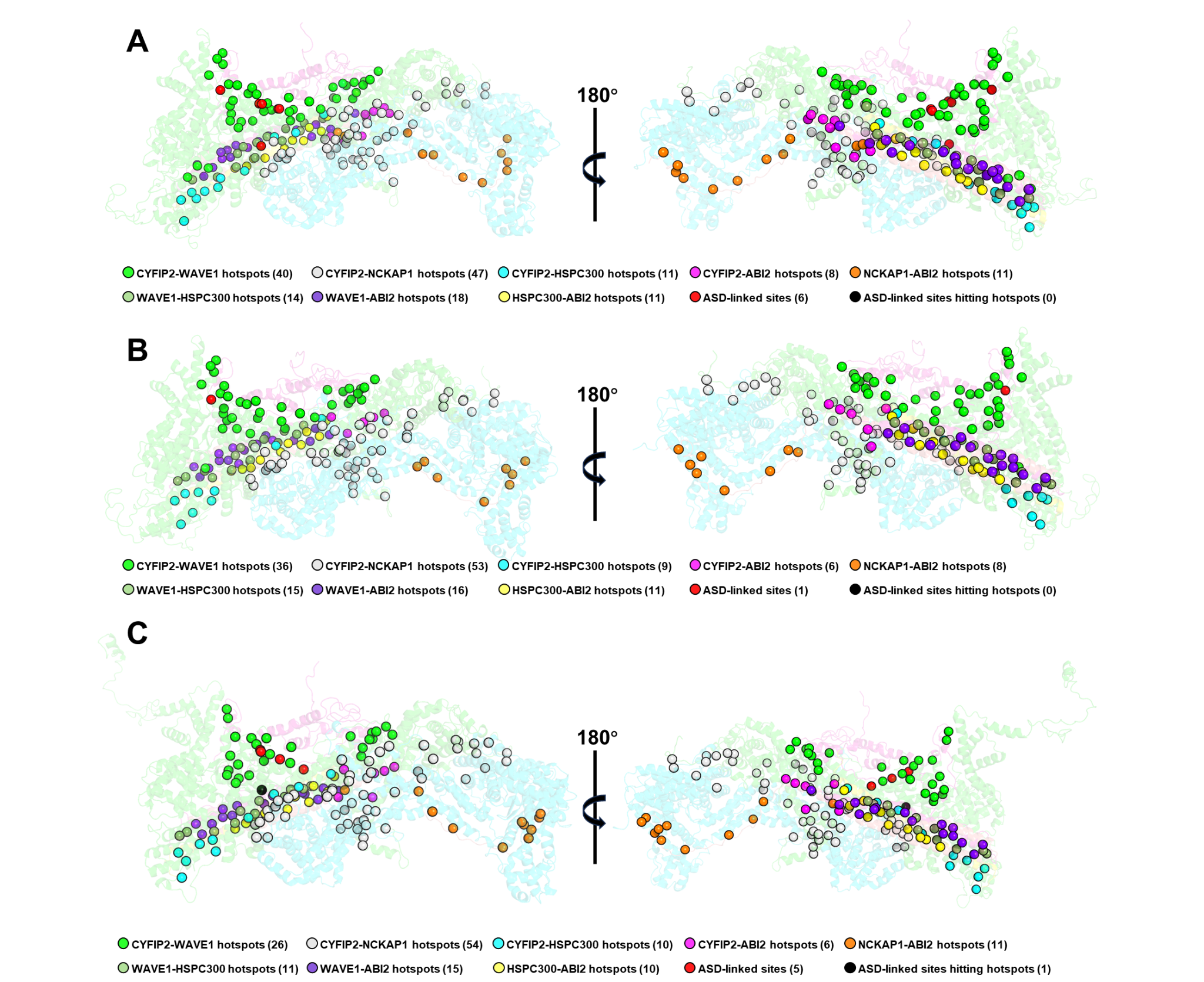
**

**Fig. S6. (A-C) Hotspots prediction of C2_iso1-3, WT_·WRC by BeAtMuSiC (Dehouck *et al.* 2013).** The color scheme is as in **Fig. 3.**

**
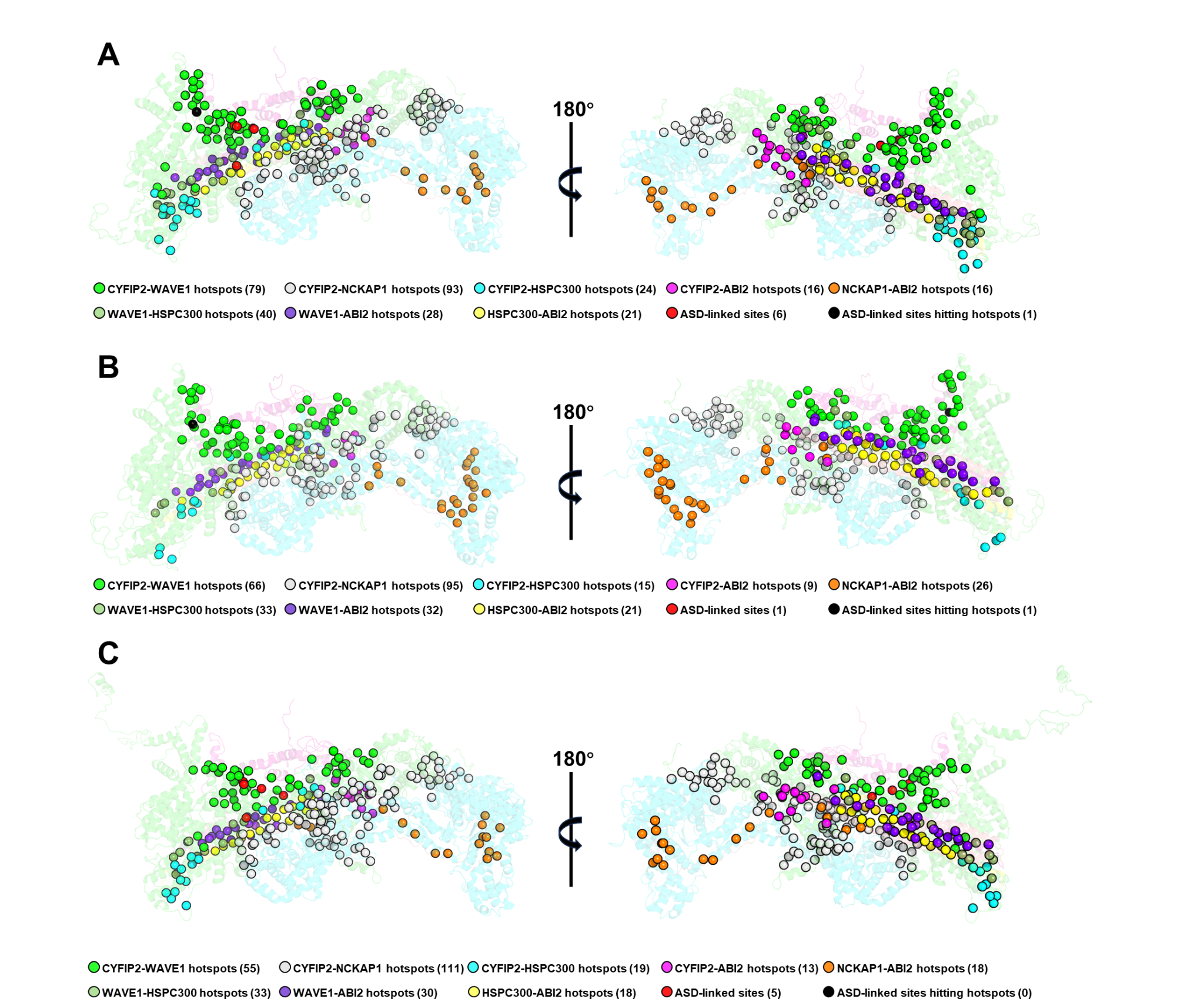
**

**Fig. S7. (A-C) Hotspot predictions of C2_iso1-3, WT_·WRC by FoldX (Schymkowitz *et al.* 2005).** The color scheme is as in **Fig. 3.**


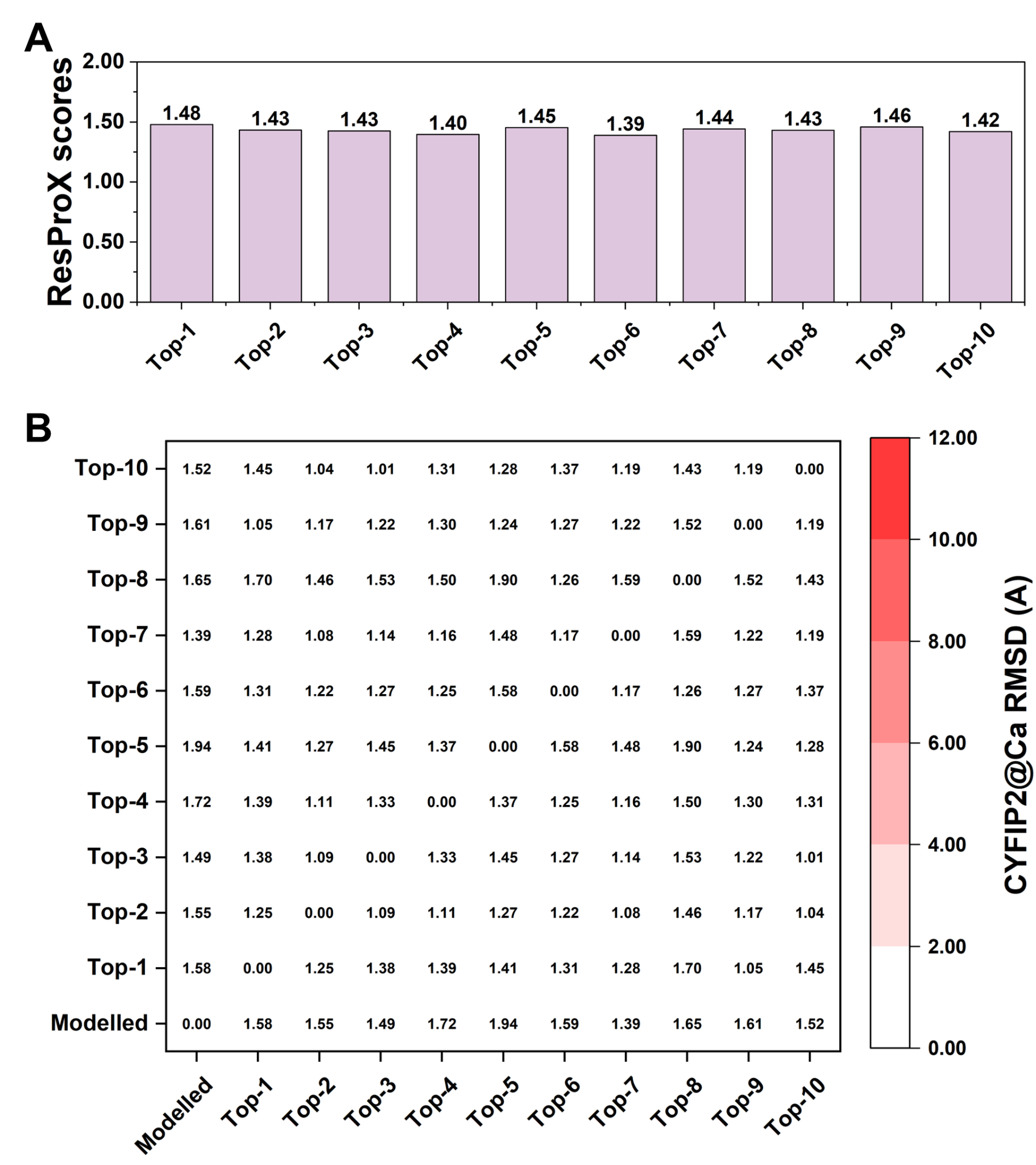


**Fig. S8.** **The performance of our protocol to predict C2_iso1, WT_·WRC structural determinants.** (A) ResProx scores of our predicted models. A ResProx score greater than 2.5 indicates a bad structure, between 1.5 and 2.5, a moderate structure, and less than 1.5, a good structure (Berjanskii *et al.* 2012). (B) The CYFIP2 Cα RMSD values between the structures obtained by our protocol and SWISS-MODEL/Modeller.


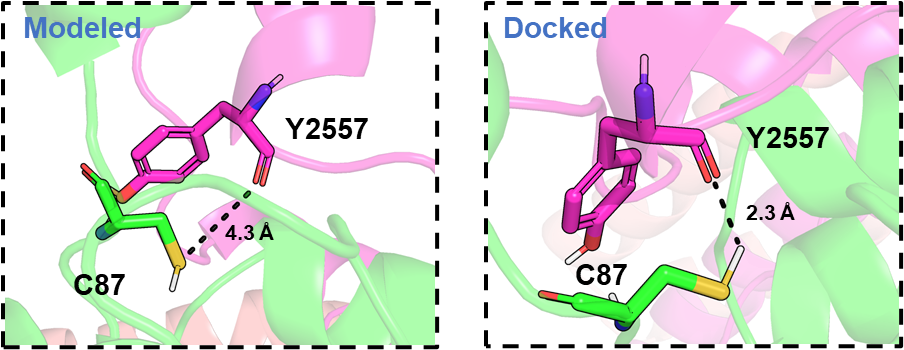


**Fig. S9. The interaction patterns of C87 variants from a standard SwissProt/Modeller procedure (left) and our protocol (right).** The H-bond is shown in the black dash.

**
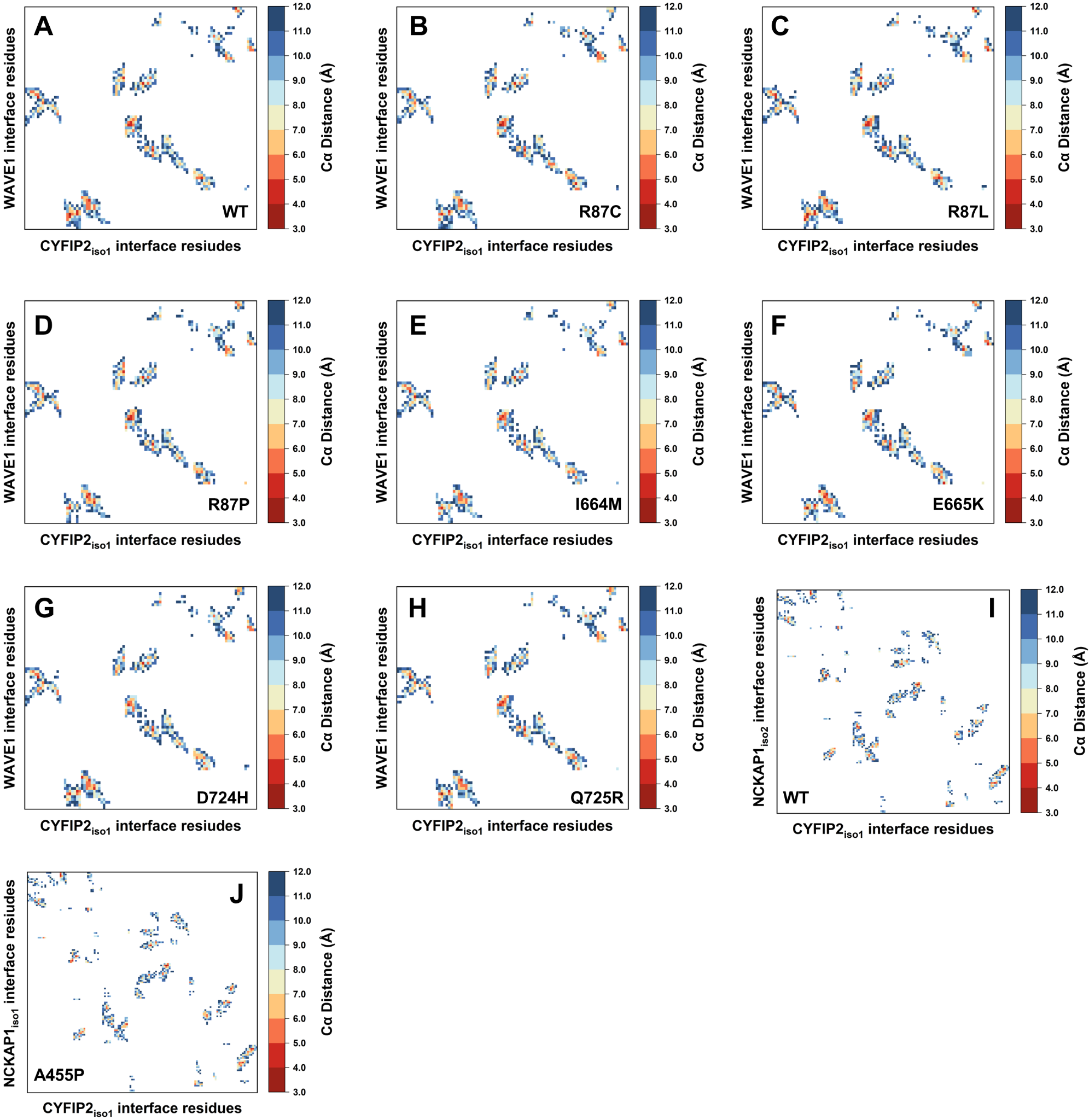
**

**Fig. S10. Contact maps of (A-H) WAVE1/CYFIP2_iso1_ and (I-J) NCKAP1_iso1_/CYFIP2_iso1_ interfaces** **in the wild-type complex and the ASD-linked variants (see Tab. 1).**

**
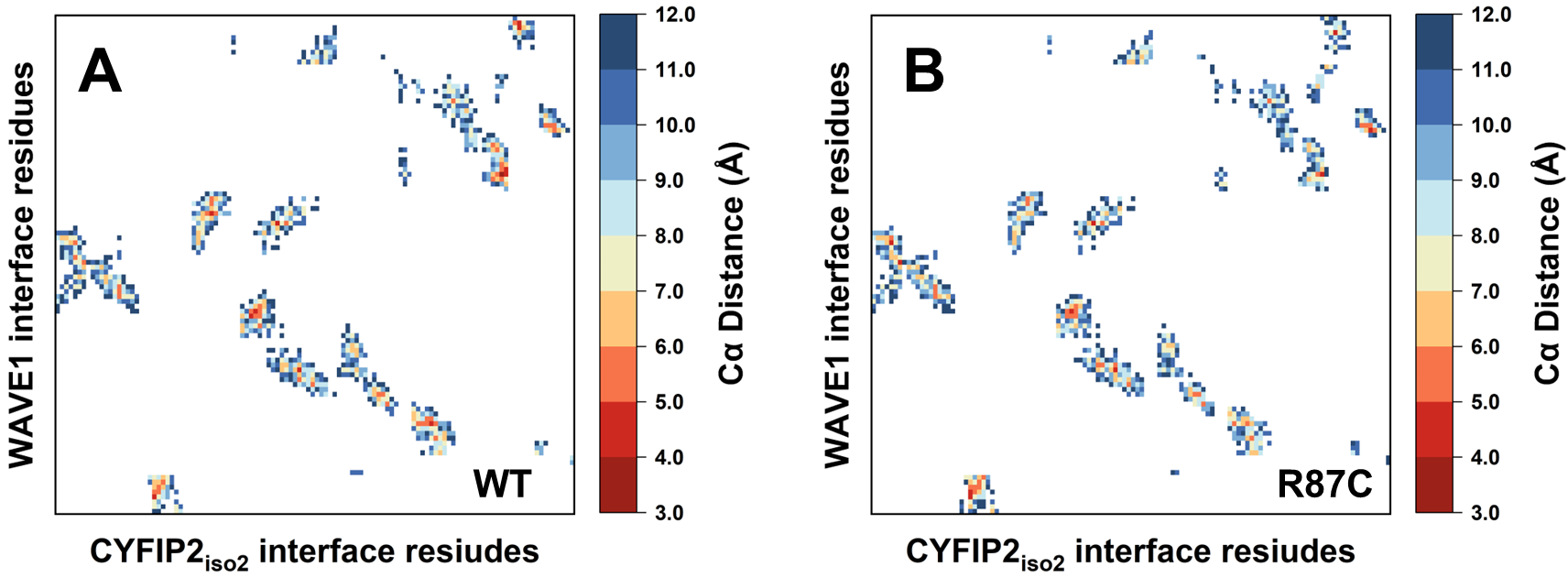
**

**Fig. S11 Contact maps of wild-type and R87C WAVE1/CYFIP2_iso2_ interfaces.**

**
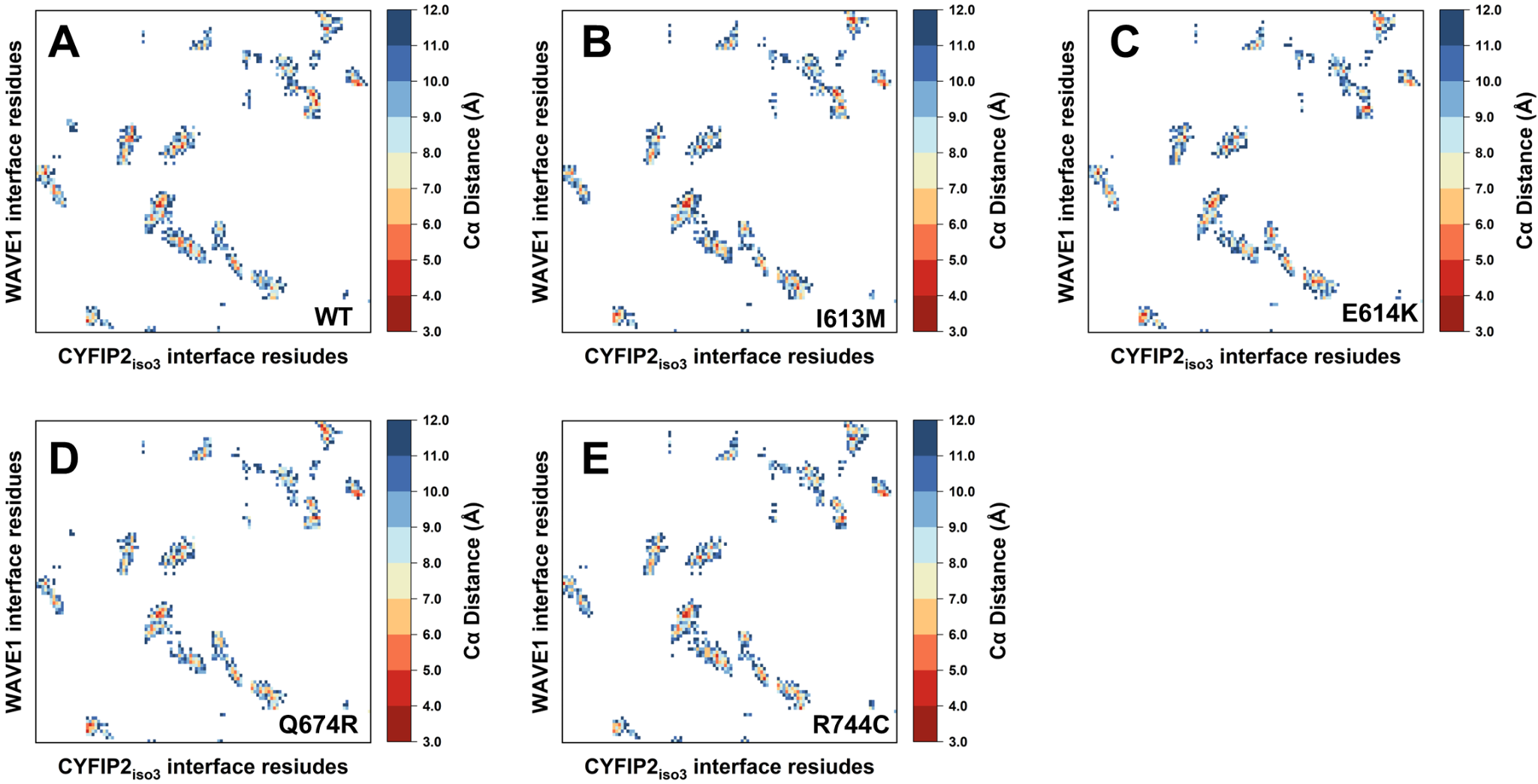
**

**Fig. S12. Contact maps of WAVE1/CYFIP2_iso3_ interface in the wild-type complex and ASD-linked variants (See Tab. 1).**

**
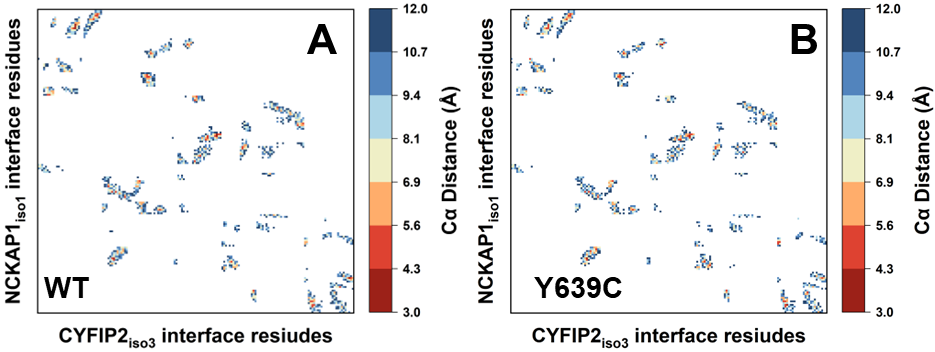
**

**Fig. S13. Contact maps of NCKAP1_iso1_/CYFIP2_iso3_ interface in the wild-type complex and ASD-linked variants (See Tab. 1).**

**
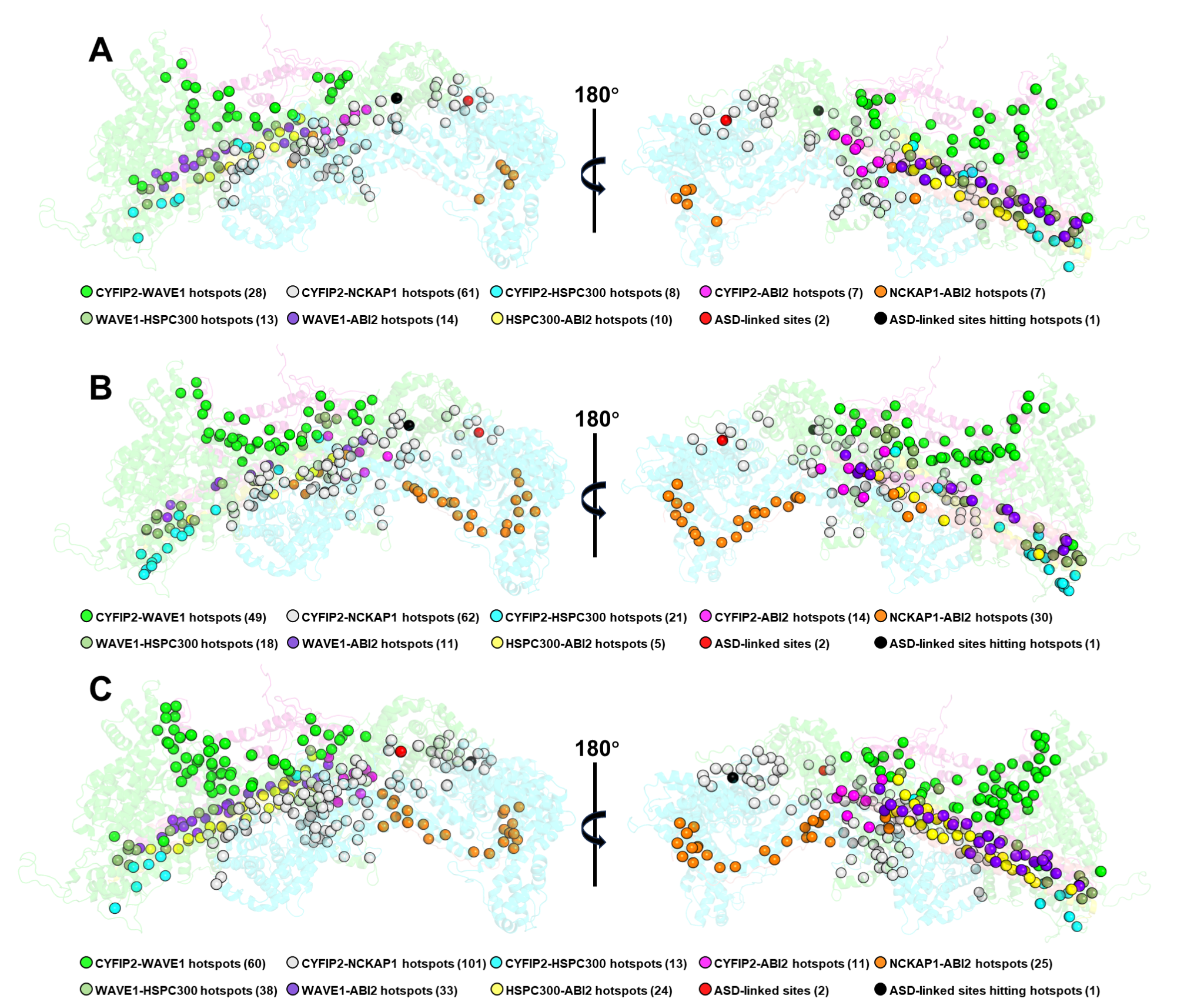
**

**Fig. S14. (A-C) Hotspots prediction of N1_iso2, WT_·WRC by (A) mCSM, (B) BeAtMuSiC, and (C) FoldX.** The color scheme is as in **Fig. 3.**

**
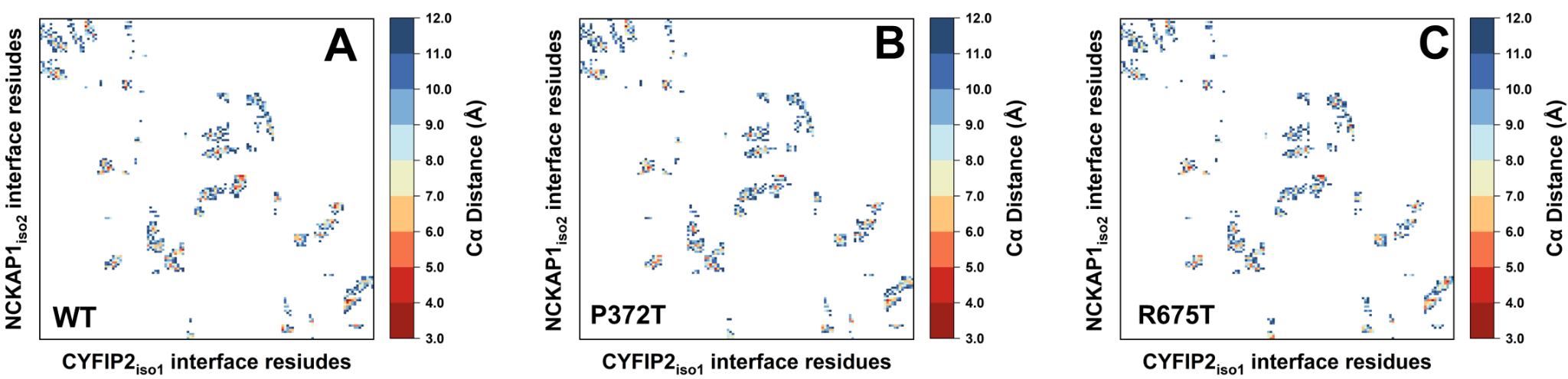
**

**Fig. S15. Contact maps of NCKAP1_iso2_/CYFIP2_iso1_ interface in the wild-type complex and ASD-linked variants (See Tab. 1).**

**
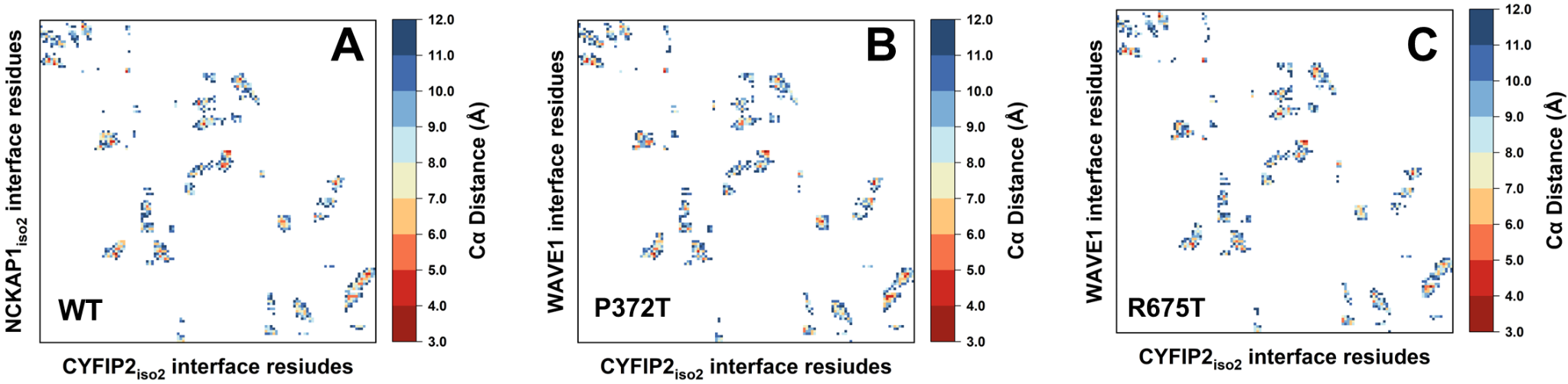
**

**Fig. S16. Contact maps of NCKAP1_iso2_/CYFIP2_iso2_ interface in the wild-type complex and ASD-linked variants (See Tab. 1).**

**
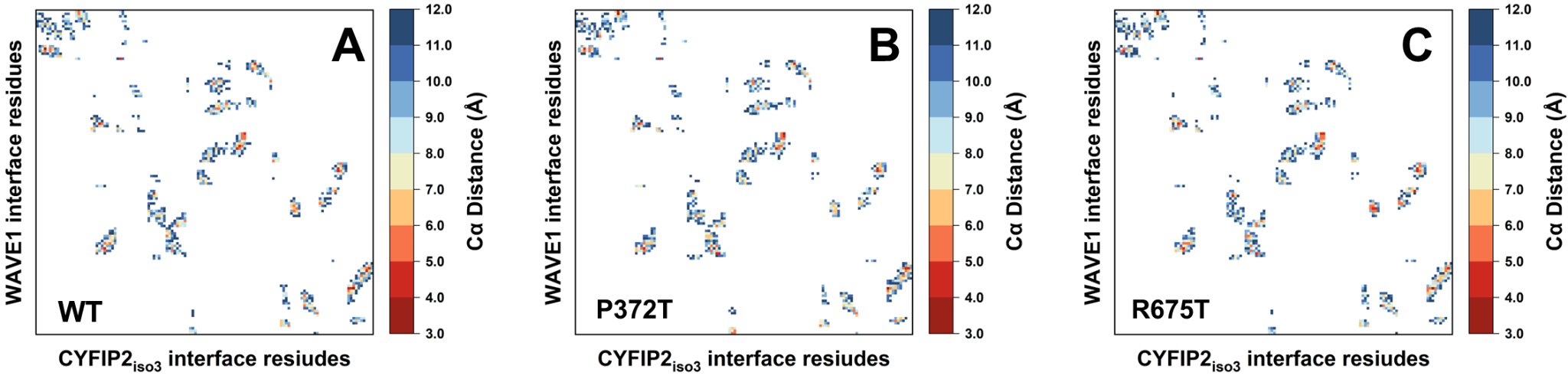
**

**Fig. S17. Contact maps of NCKAP1_iso2_/CYFIP2_iso3_ interface in the wild-type complex and ASD-linked variants (See Tab. 1).**


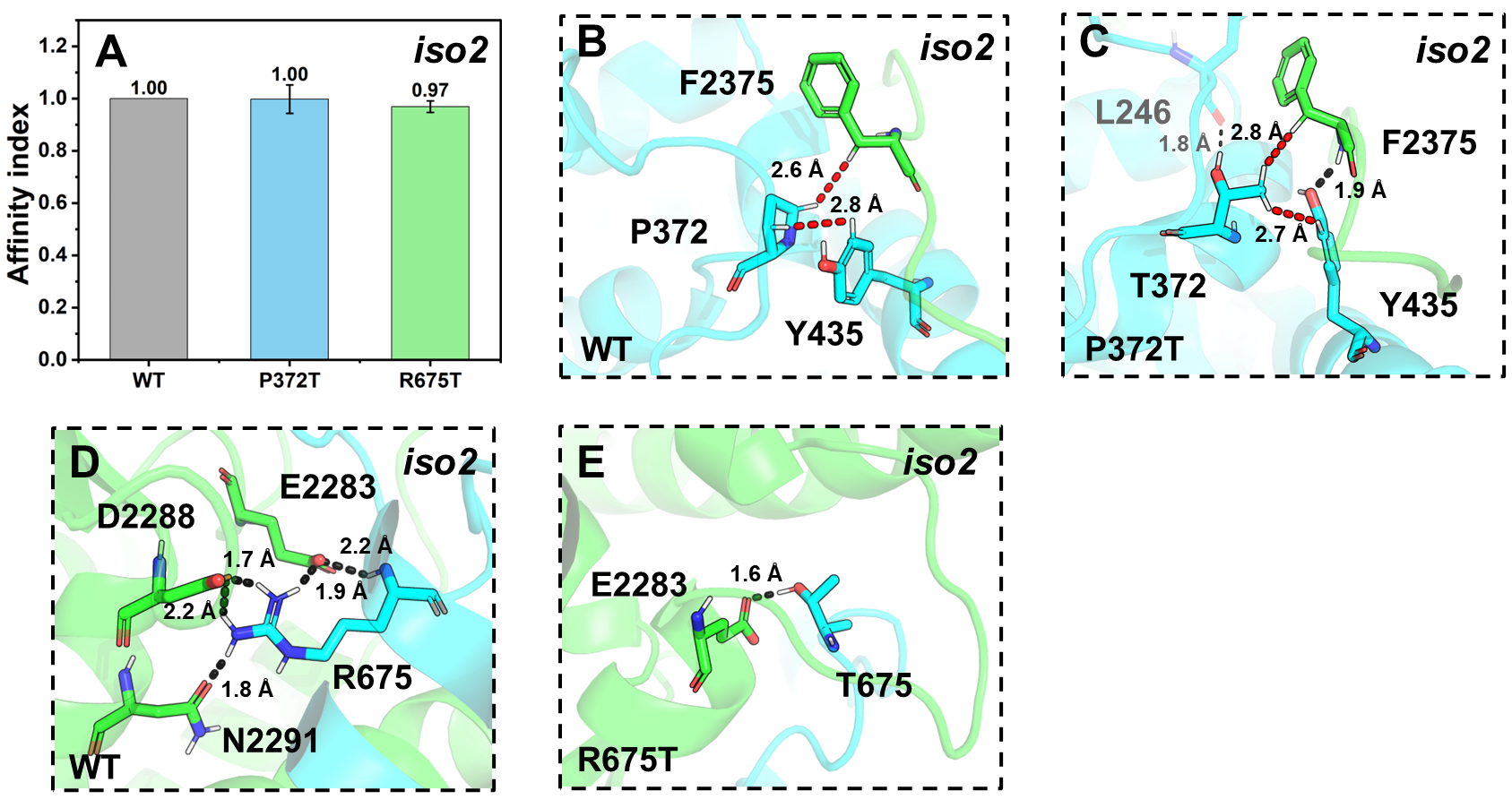


**Fig. S18. ASD-related mutations at the CYFIP2_iso2_/NCKAP1_iso2_ interface.** (A) The affinity index (values are represented as mean ± SD (N = 3)) and interaction alterations in the variants associated with (B-C) P372T (D-E) R675T.


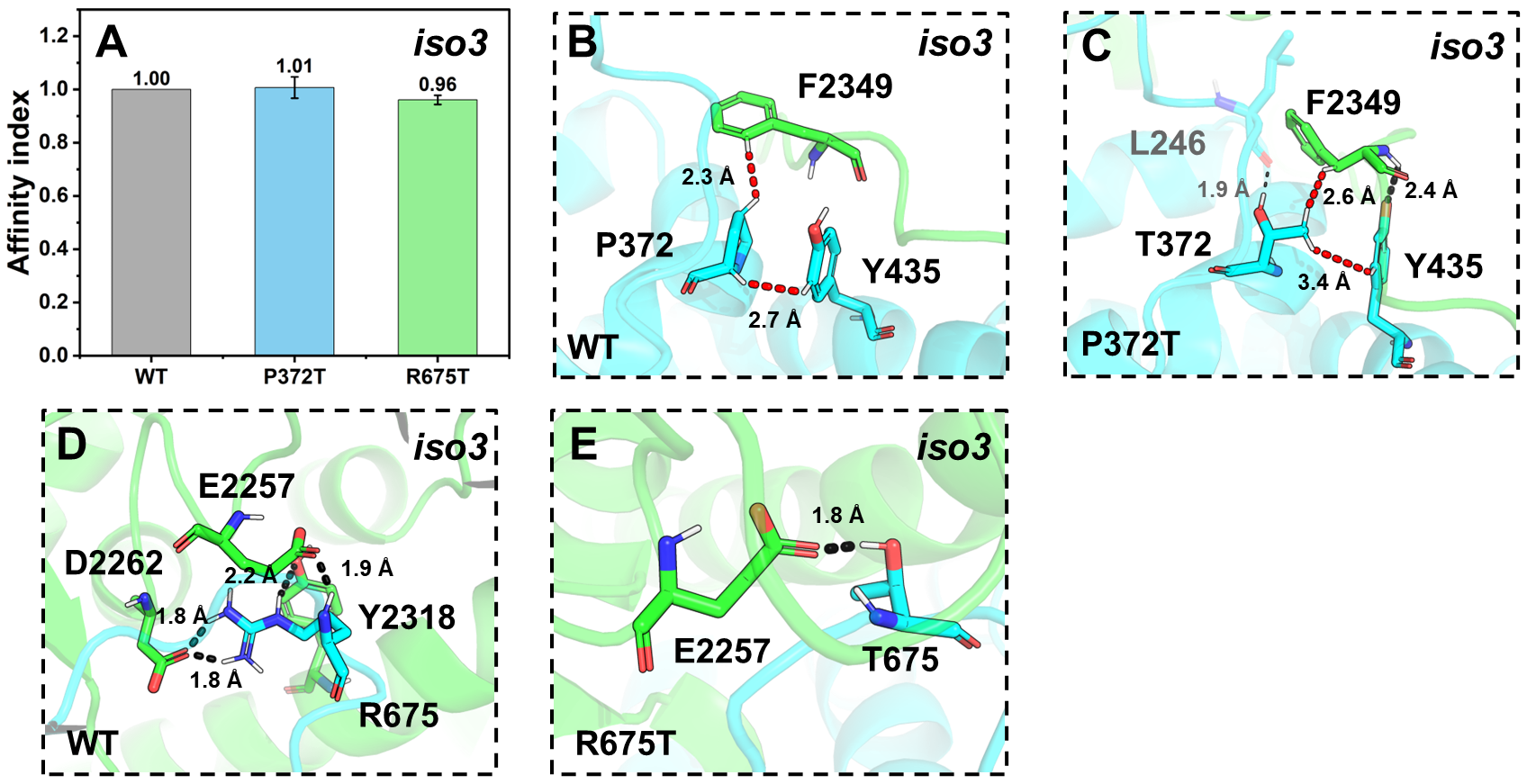


**Fig. S19. ASD-related mutations at the CYFIP2_iso3_/NCKAP1_iso2_ interface.** (A) The affinity index (values are represented as mean ± SD (N = 3)) and interaction network in the variants associated with (B-C) P372T, (D-E) R675T.


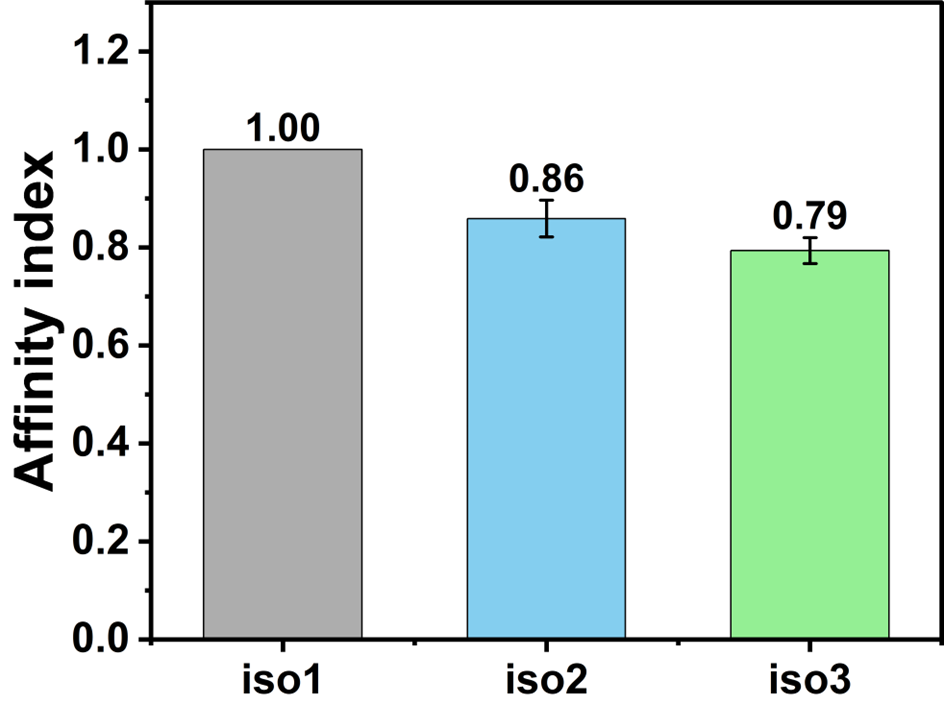


**Fig. S20.** **Affinity indexes of the wild-type CYFIP2_iso1-3_/WAVE1 interfaces.** Values are represented as mean ± SD (N = 3).


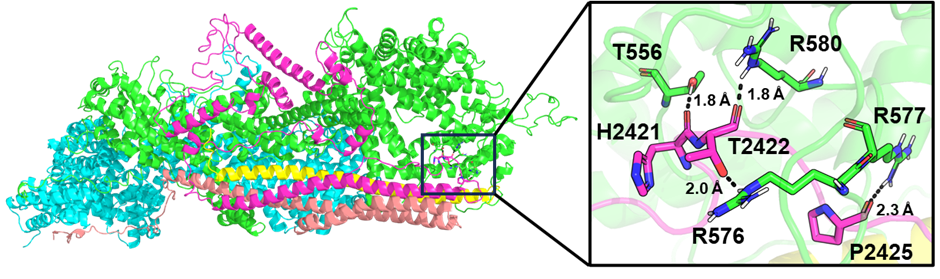


**Fig. S21. Details of missing region 1 (MR1, residues 558-582) and WAVE1 interaction.** The CYFIP2_iso1_, NCKAP1_iso1_, WAVE1, HSPC300, and ABI2 are shown in **Fig. 2**. In the inset, the interacting residues and the intermolecular interactions are represented in **Fig. 5**. are highlighted using sticks, and black dashes represent the hydrogen bonds.

**4. Supplementary Tables**

**Tab. S1 ASD-linked variants of CYFIP2_iso1-3._**

| **Uniprot ID** | **Variant** | **Reference** |
| --- | --- | --- |
| **Q96F07-1**  **(iso1, canonical)** | p.Arg87Cys | (Nakashima *et al.* 2018) |
|  | p.Arg87Leu | (Nakashima *et al.* 2018) |
|  | p.Arg87Pro | (Nakashima *et al.* 2018) |
|  | p.Tyr108His | (Zweier *et al.* 2019) |
|  | p.Ala455Pro | (Zweier *et al.* 2019) |
|  | p.Ile664Met | (Zweier *et al.* 2019) |
|  | p.Glu665Lys | (Zweier *et al.* 2019) |
|  | p.Asp724His | (Zweier *et al.* 2019) |
|  | p.Gln725Arg | (Zweier *et al.* 2019) |
| **Q96F07-2**  **(iso2)** | p.Val14Met | (Firth *et al.* 2009) |
|  | p.Arg87Cys | (Firth *et al.* 2009) |
|  | p.Val181Phe | (Firth *et al.* 2009) |
|  | p.Ala481Val | (Fu *et al.* 2022) |
| **E7EVJ5**  **(iso3, computationally mapped)** | p.Ile613Met | (Firth *et al.* 2009) |
|  | p.Glu614Lys | (Firth *et al.* 2009) |
|  | p.Tyr639Cys | (Firth *et al.* 2009) |
|  | p.Gln674Arg | (Firth *et al.* 2009) |
|  | p.Arg744Cys | (Firth *et al.* 2009) |

**Tab. S2 ASD-linked variants of NCKAP1_iso1-2._**

| **Uniprot ID** | **Variants** | **Reference** |
| --- | --- | --- |
| **Q9Y2A7-1**  **(iso1, canonical)** | p.Asp323Asn | (Li *et al.* 2022) |
|  | p.Asp538His | (Wang *et al.* 2020) |
| **Q9Y2A7-2**  **(iso2)** | p.Ser2Trp | (Guo *et al.* 2020) |
|  | p.Ile143Phe | (Sheth *et al.* 2023) |
|  | p.Arg349Cys | (Wang *et al.* 2020) |
|  | p.Pro372Thr | (Wang *et al.* 2020) |
|  | p.Val437Met | (Wang *et al.* 2020) |
|  | p.Phe489Ile | (Wang *et al.* 2020) |
|  | p.Ala513Thr | (Guo *et al.* 2020) |
|  | p.Glu541Lys | (Wang *et al.* 2020) |
|  | p.Asp544His | (Wang *et al.* 2020) |
|  | p.Arg675Thr | (Wang *et al.* 2020) |
|  | p.Thr679Ser | (Firth *et al.* 2009) |
|  | p.Pro710Leu | (Wang *et al.* 2020) |
|  | p.Ala1121Val | (Guo *et al.* 2020) |

**Tab. S3.** **Cα RMSD values between the C2_iso1-3, WT_·WRC model obtained by SWISS-MODEL/Modeller procedure and its X-ray structure (PDB ID: 3P8C (Chen *et al.* 2010)).** The RMSD values were evaluated by PyMOL 2.5.2 (Schrödinger, L. 2020).

|  | **RMSD (**Å**)** |
| --- | --- |
| **CYFIP2_iso1_·WRC** | 0.9 |
| **CYFIP2_iso2_·WRC** | 0.6 |
| **CYFIP2_iso3_·WRC** | 0.9 |

**Tab. S4. Sequence identity and similarity between CYFIP1_iso1_ and CYFIP2_iso1-3_. The values have been obtained using MUSCLE (Edgar 2004).**

|  | **Uniprot ID** | **Length** | **Identity** | **Similarity** |
| --- | --- | --- | --- | --- |
| **CYFIP2_iso1_** | Q96F07-1 | 1278 | 86% | 92% |
| **CYFIP2_iso2_** | Q96F07-2 | 1253 | 88% | 94% |
| **CYFIP2_iso3_** | E7EVJ5 | 1227 | 86% | 92% |

**Tab. S5. Interface residues of C2_iso1, WT_·WRC, by visual inspection on our predicted models.**

| **Interface** | **CYFIP2_iso1_** | **Partner** |
| --- | --- | --- |
| **CYFIP2_iso1_-NCKAP1_iso1_** | 353-354, 355-357, 360-362, 364-365, 368, 445, 448, 452, 456-457, 463, 466, 683-684, 685-686, 690, 737, 741, 745, 748-751, 755, 758,760, 764, 767-769, 775, 827, 830, 834, 838-839, 882-885, 887, 889-890, 921, 924-925, 927-929, 931-932, 943, 988-990, 1032, 1036, 1039-1040, 1043-1052, 1055-1061, 1077-1078, 1080-1081, 1083, 1086, 1089-1090, 1095, 1099, 1106, 1111-1113, 1140, 1142-1143, 1145-1146, 1172, 1174-1175, 1178-1179, 1182, 1209, 1213, 1231, 1233-1235, 1237-1238, 1240-1241, 1245, 1248-1249, 1251, 1260, 1263-1271, 1273-1274 | 1503-1506, 1511, 1514, 1517-1518, 1619, 1621, 1626, 1629-1630, 1633-1634, 1636-1641, 1699, 1703, 1706-1707, 1710-1711, 1714-1715, 1718-1719, 1842, 1899, 1902, 1906-1908, 1911-1913, 1915-1916, 1937-1947, 1950, 1953-1954, 1957-1958, 1961-1962, 2013, 2025-2026, 2029-2030, 2032-2036, 2038, 2040-2041, 2043-2044, 2047-2049, 2052-2057, 2088-2090, 2106-2107, 2110-2112, 2115, 2118-2119, 2122-2124, 2127, 2131, 2165, 2204, 2206, 2208-2210, 2216-2217, 2219-2220, 2232, 2235-2236, 2238-2239, 2242-2243, 2296, 2298, 2301, 2338, 2342, 2345-2346, 2348-2349, 2351, 2353, 2359, 2363, 2367, 2371, 2378, 2381, 2386-2387, 2389, 2391-2392, 2394-2395, 2398-2399 |
| **CYFIP2_iso1_-WAVE1** | 85-89, 91-94, 97, 103, 106-107, 110, 114, 121, 439, 516, 555-560, 574-577, 579-582, 630-631, 633-634, 648, 659-665, 703, 707-710, 712-714, 716-718, 720-721, 723-725, 728, 781-784, 786, 790-791, 794-795, 798, 801, 805, 812, 851, 854, 856-860, 862, 865, 868-869, 872-873, 900-903, 906, 908-910, 948, 956, 960, 963, 1023 | 2415-2418, 2420-2426, 2431, 2434-2435, 2496, 2498, 2500-2501, 2506-2509, 2512-2519, 2522-2524, 2527, 2530-2532, 2534-2537, 2539-2540, 2543, 2546-2548, 2553, 2556-2560, 2562-2564, 2566-2567, 2570-2571, 2609-2610, 2619-2620, 2624-2628, 2631, 2635, 2638-2639, 2657-2658, 2661-2662, 2665, 2669-2670 |
| **CYFIP2_iso1_-HSPC300** | 468-469, 472-473, 476, 480, 484, 514-520, 523-529, 532, 542, 549-554, 777-778, 780, 787, 789-790, 840-841, 843, 846-847, 850, 856-857 | 2686-2691, 2694-2695, 2697-2698, 2701-2702, 2704-2705, 2708, 2715-2716, 2726, 2729-2730, 2733-2734, 2736-2737, 2740, 2744, 2748, 2751-2752, 2756 |
| **CYFIP2_iso1_-ABI2** | 850, 947-948, 951, 955, 1092, 1094-1095, 1098, 1101, 1105-1106, 1108-1111, 1113-1116, 1118-1120, 1123, 1251 | 2852, 2855-2856, 2858-2859, 2862-2863, 2865-2866, 2869, 2872-2877 |

**Tab. S6. Interface residues of C2_iso2, WT_·WRC, by visual inspection on our predicted models.**

| **Interface** | **CYFIP2_iso2_** | **Partner** |
| --- | --- | --- |
| **CYFIP2_iso2_-NCKAP1_iso1_** | 353-354, 356-358, 360-362, 364-365, 368, 371, 445, 452, 455-456, 459, 463, 466, 658-659, 661-662, 665, 720, 723-726, 729, 733, 735, 742, 744, 747, 749, 802, 805, 809, 813-814, 857-862, 864-865, 896, 899, 903-904, 906-907, 910, 918, 963-965, 1007, 1011, 1012, 1014-1016, 1018-1027, 1030-1036, 1052-1053, 1055-1056, 1058, 1061, 1065-1066, 1070, 1086-1088, 1115, 1117-1118, 1120-1121, 1147, 1149-1150, 1154, 1157, 1168, 1171-1172, 1174-1175, 1184, 1187-1188, 1202, 1206, 1208-1210, 1212-1213, 1215-1216, 1220, 1223-1224, 1235, 1238-1246, 1249 | 1478-1481, 1489, 1492-1493, 1495, 1594, 1597, 1604-1605, 1608-1609, 1611-1616, 1675, 1678, 1681-1682, 1685-1686, 1688-1690, 1693, 1817, 1877, 1881-1884, 1886-1887, 1889-1891, 1893, 1912-1923, 1925, 1928-1929, 1932-1933, 1936-1937, 1943, 1988, 1997, 2000-2001, 2004-2005, 2007-2011, 2013, 2015-2016, 2018-2019, 2022-2024, 2027-2033, 2063-2065, 2081-2082, 2085-2087, 2090, 2093-2094, 2097-2099, 2102, 2106, 2175, 2179, 2181-2185, 2192, 2193, 2194, 2207, 2210-2211, 2213-2214, 2217, 2273, 2276, 2313, 2317, 2320, 2323-2324, 2334, 2338, 2342, 2346, 2353, 2357-2358, 2361-2364, 2366-2367, 2369-2370, 2373-2374 |
| **CYFIP2_iso2_-WAVE1** | 85-87, 89, 91-94, 97, 103, 105-106, 110, 114, 121, 439, 554-557, 605-606, 608-609, 612, 623, 634-638, 640, 678, 682-685, 687-689, 691, 693-696, 698-700, 703, 707, 756-759, 761, 765-766, 769-770, 773, 776, 784, 787, 826-827, 829, 831-835, 837, 840, 843-844, 847-848, 875-878, 881-883, 885, 923, 931, 935, 998 | 2661-2666, 2669-2670, 2672-2673, 2675-26777, 2679-2680, 2683, 2687, 2690-2691, 2674, 2697, 2701, 2704-2705, 2708-2709, 2711-2712, 2719, 2723, 2726-2727 |
| **CYFIP2_iso2_-HSPC300** | 466, 468-469, 472-473, 476, 480, 514-517, 519-520, 524-529, 532, 542, 549-554, 557, 608, 752-753, 755, 760, 762, 764-765, 776, 816, 818, 822, 825-826, 831-832 | 2686-2691, 2694-2695, 2697-2698, 2701-2702, 2704-2705, 2708, 2715-2716, 2726, 2729-2730, 2733-2734, 2736-2737, 2740, 2744, 2748, 2751-2752, 2756 |
| **CYFIP2_iso2_-ABI2** | 825, 922-923, 925-926, 1070, 1073, 1076, 1080, 1083-1084, 1086, 1088-1091, 1093-1094, 1098 | 2827, 2830-2831, 2833-2834, 2837-2838, 2840-2841, 2844, 2847-2851 |

**Tab. S7. Interface residues of C2_iso3, WT_·WRC, by visual inspection on our predicted models.**

| **Interface** | **CYFIP2_iso3_** | **Partner** |
| --- | --- | --- |
| **CYFIP2_iso3_-NCKAP1_iso1_** | 327, 330-332, 334-336, 338-339, 342, 419, 426, 429-430, 437, 440, 631-633, 635-636, 639, 690, 694, 697-700, 703-704, 707, 709, 713, 716, 718, 721, 724, 772, 776, 779-780, 783, 787-788, 831-834, 836, 838-839, 870, 873-874, 876-878, 880-881, 892, 937-939. 981, 985, 988-989, 992, 994, 996-1001, 1004-1009, 1026-1027, 1029-1030, 1032, 1035, 1038-1040, 1044, 1055, 1060-1062, 1089, 1091-1092, 1094, 1121, 1123-1124, 1127-1128, 1142, 1145-1146, 1148-1149, 1158, 1162, 1180, 1182-1185, 1186-1187, 1189-1190, 1194, 1197-1198, 1200, 1212-1220, 1223 | 1452-1455, 1463, 1466-1467, 1469, 1568, 1570, 1575, 1579, 1582-1583, 1585-1590, 1655-1656, 1659-1660, 1662-1664, 1667-1668, 1787-1788, 1791, 1848, 1851-1852, 1855-1858, 1860-1865, 1867-1869, 1886-1888, 1890-1897, 1899, 1902-1903, 1906-1907, 1910-1911, 1917, 1962, 1974-1975, 1978-1979, 1981-1985, 1989-1990, 1992-1993, 1996-1998, 2001-2005, 2037-2039, 2055-2056, 2059-2060, 2064, 2067-2068, 2071-2073, 2076, 2080, 2149, 2153-2155, 2157-2159, 2168-2169, 2181, 2184, 2187-2188, 2191, 2247, 2287, 2291, 2294, 2297-2298, 2300, 2312, 2316, 2320, 2331-2333, 2335-2338, 2340-2341, 2343-2345, 2347-2348 |
| **CYFIP2_iso3_-WAVE1** | 80-81, 84-85, 88, 95, 413, 528-531, 579-583, 597, 608-614, 652, 656-659, 661-663, 665-670, 672-674, 677, 730-733, 739-740, 743-744, 747, 750, 761, 800-801, 803, 805-809, 811, 814, 817-818, 821-822, 849-852, 855-857, 859, 897, 905, 908-909 | 2366-2367, 2369-2370, 2373, 2445-2447, 2450, 2455-2458, 2461-2469, 2471-2473, 2476, 2479-2481, 2483-2489, 2491-2492, 2495, 2504-2409, 2511-2512, 2515-2516, 2519, 2558-2560, 2566, 2568-2570, 2573-2577, 2580, 2587, 2607, 2610-2611, 2614, 2618-2619 |
| **CYFIP2_iso3_-HSPC300** | 442-443, 446-447, 450, 458, 488-494, 497-503, 506, 516, 523-527, 582, 726-728, 739, 750, 790, 792, 795-797, 799, 805-806 | 2635-2640, 2642-2644, 2646-2647, 2650-2651, 2653-2654, 2657, 2664-2665, 2668, 2675, 2678-2679, 2682-2683, 2685-2686, 2689-2690, 2693, 2697, 2700-2701, 2703, 2705 |
| **CYFIP2_iso3_-ABI2** | 799, 809, 896-897, 899-900, 903, 1043-1044, 1047, 1050, 1054-1055, 1057-1059, 1062-1065, 1067-1068, 1072, 1200-1201 | 2797, 2781, 2804-2805, 2807-2808, 2811-2812, 2814-2815, 2817-2818, 2820-2826 |

**Tab. S8.** **The Cα RMSD values among our predicted model of N1_iso2, WT_·WRC and a model obtained by SWISS-MODEL/Modeller.** The RMSD values were evaluated by PyMOL 2.5.2.

|  | **RMSD (Å)** |
| --- | --- |
| **NCKAP1_iso2_·WRC with CYFIP2_iso1_** | 2.1 |
| **NCKAP1_iso2_·WRC with CYFIP2_iso2_** | 2.0 |
| **NCKAP1_iso2_·WRC with CYFIP2_iso3_** | 2.5 |

**Tab. S9. Unprot ID of the protein components of the 3P8C X-ray structure (Chen *et al.* 2010).**

| **Protein** | **Uniprot ID** |
| --- | --- |
| **CYFIP1** | Q7L576-1 |
| **NCKAP1** | Q9Y2A7-1 |
| **WAVE1** | Q92558 |
| **HSPC300** | Q8WUW1-1 |
| **ABI2** | Q9NYB9-1 |

**Tab. S10.** **Cα RMSD (Å)** **values among the top-rated predicted C2_iso1-3, WT_·WRC complexes.** The RMSD values were evaluated by PyMOL 2.5.2 (Schrödinger, L. 2020).

|  | **CYFIP2_iso1_** | **CYFIP2_iso2_** | **CYFIP2_iso3_** |
| --- | --- | --- | --- |
| **CYFIP2_iso1_** | 0.0 | 1.3 | 1.7 |
| **CYFIP2_iso2_** | 1.3 | 0.0 | 1.7 |
| **CYFIP2_iso3_** | 1.7 | 1.7 | 0.0 |

**Tab. S11. Interface residues of N1_iso2, WT_·WRC with CYFIP2**_iso1_ **by visual inspection on our predicted models.**

| **Interface** | **NCKAP1_iso2_** | **Partner** |
| --- | --- | --- |
| **NCKAP1_iso2_-CYFIP2_iso1_** | 231-232, 234, 239, 242, 245-248, 350-351, 354, 357-358, 361-362, 364-369, 372, 410, 413, 428, 431, 435, 438-439, 442-443, 446, 450, 567, 570, 627, 631, 634-637, 639, 641-644, 647, 650, 653, 655, 657, 665-667, 669-676, 678, 681-682, 685-686, 689-690, 693, 696-697, 699, 719, 741, 750, 753-754, 757-758, 760-762, 764, 768-769, 771-773, 775-777, 779-782, 784, 786, 817-818, 832, 834-835, 838-841, 843, 847, 850-852, 859, 891-894, 928, 931-941, 944-948, 956, 960, 963, 966-967, 1026, 1029, 1070, 1073, 1076-1077, 1079, 1081, 1087, 1095, 1099, 1106, 1110-1112, 1114-1117, 1119-1120, 1122-1123, 1126-1127 | 1487-1488, 1490-1492, 1494-1496, 1499, 1502-1503, 1506, 1519, 1522, 1579, 1593, 1596-1597, 1600, 1815-1817, 1820-1821, 1824, 1871, 1875, 1878-1880, 1882-1886, 1888-1890, 1892-1894, 1896, 1898-1901, 1903, 1905-1906, 1909-1910, 1957-1958, 1960-1961, 1964-1965, 1968-1969, 1972-1973, 1980, 1983, 2016-2021, 2023, 2054, 2058-2059, 2061-2063, 2065-2066, 2069-2070, 2073, 2077, 2120, 2122-2124, 2169-2170, 2173-2182, 2185-2186, 2189, 2191-2195, 2204, 2208, 2211-2212, 2215, 2217, 2220-2221, 2223-2224, 2229, 2232, 2236-2237, 2239-2240, 2243, 2245-2246, 2274-2277, 2279-2280, 2300-2303, 2306, 2308-2309, 2313, 2330-2331, 2333-2335, 2339, 2343, 2346-2347, 2364-2365, 2367-2368, 2371, 2374-2375, 2379, 2381-2383, 2385, 2390, 2393-2394, 2396-2398, 2400-2405, 2407-2408, 2412 |
| **NCKAP1_iso2_-WAVE1** | 654 | 2602 |
| **NCKAP1_iso2_-ABI2** | 12, 15-16, 18-20, 23, 30, 105, 108, 112, 115, 464-465, 468, 490-491, 494-495, 498, 588-589, 591-592, 594-595, 606, 704-709, 711-713, 716, 719, 760, 764-766, 940, 943-944, 947, 950-951, 954 | 2843, 2847, 2850, 2854, 2857-2858, 2861, 2864-2865, 2868, 2882-2887, 2889-2891, 2893, 2896-2902, 2904-2909, 2911-2919 |

**Tab. S12. Interface residues of N1_iso2, WT_·WRC with CYFIP2**_iso2_ **by visual inspection on our predicted models.**

| **Interface** | **NCKAP1_iso2_** | **Partner** |
| --- | --- | --- |
| **NCKAP1_iso2_-CYFIP2_iso2_** | 228, 231-232, 234, 239, 242, 245-246, 248, 350, 354, 357-358, 361-362, 364-369, 410, 413, 427-428, 431, 434-435, 438-439, 442-443, 446, 450, 478, 567, 570, 627, 630-631, 634, 636-637, 639, 641-644, 647-649, 651, 660, 665-676, 679, 681-682, 685-686, 689-690, 693, 696, 741, 743, 750, 753-754, 757, 760-762, 764, 768-769, 771-773, 775-777, 780-786, 817-818, 832, 834-835, 838-841, 843, 847, 850-852, 855-856, 859, 891, 893-894, 928, 931-932, 934-938, 940-941, 944-945, 947-948, 951-952, 960, 963, 966-967, 1026, 1029, 1073, 1076-1077, 1079, 1087, 1091, 1095, 1099, 1106, 1110-1111, 1114-1117, 1119-1120, 1122-1123, 1126-1127 | 1487-1488, 1490-1492, 1494-1496, 1499, 1502, 1579, 1586, 1593, 1597, 1600, 1790-1792, 1795-1796, 1799, 1846, 1850, 1854-1855, 1857-1861, 1863-1865, 1867, 1869, 1871, 1873-1876, 1878, 1880-1881, 1885, 1932-1933, 1936, 1939-1940, 1943-1944, 1947, 1991-1996, 1998, 2025-2026, 2029, 2033-2034, 2036-2038, 2040-2041, 2044-2045, 2048, 2052, 2097-2099, 2145, 2148-2150, 2152-2157, 2160-2161, 2164, 2166-2169, 2171, 2186-2187, 2189-2190, 2192, 2195-2196, 2198-2201, 2204, 2207, 2211-2212, 2214-2215, 2220-2222, 2249, 2251-2252, 2254, 2275-2278, 2281, 2283-2284, 2288, 2305-2306, 2308-2310, 2318, 2321-2322, 2336, 2339-2340, 2342-2344, 2346, 2349-2350, 2354, 2357-2358, 2360, 2364-2365, 2369-2370, 2372-2383 |
| **NCKAP1_iso2_-WAVE1** | 654 | 2577 |
| **NCKAP1_iso2_-ABI2** | 9-10, 12, 15-16, 18-20, 23, 30, 94, 98, 101, 104-105, 108, 112, 178-179, 464-465, 468, 490-491, 494-495, 498, 502, 588-589, 591-592, 594-595, 606, 610, 704-707, 709, 712-713, 716, 719, 760, 764-766, 768-769, 940, 943-944, 947, 951 | 2829, 2832-2833, 2836, 2839-2840, 2843, 2857-2866, 2868, 2871-2884, 2886-2888, 2890-2895 |

**Tab. S13. Interface residues of N1_iso2, WT_·WRC with CYFIP2**_iso3_ **by visual inspection on our predicted models.**

| **Interface** | **NCKAP1_iso2_** | **Partner** |
| --- | --- | --- |
| **NCKAP1_iso2_-CYFIP2_iso3_** | 228, 231-232, 234, 239-240, 242-243, 245-246, 248, 350, 353-354, 357-358, 361-362, 364-369, 372, 413, 428, 431, 435, 438-439, 442-443, 446, 450, 570, 627, 630-631, 634-637, 639, 641-644, 647-648, 660, 665-668, 670-676, 678-679, 681-682, 685-686, 689-690, 693, 696, 741, 750, 753-754, 757-758, 760-762, 764, 768-769, 771-773, 775-777, 780-786, 817-818, 821, 832, 834-835, 838-841, 843, 847, 850-852, 855-856, 859, 890-891, 893-894, 928, 931-932, 934-941, 944-945, 947-948, 951-953, 960, 963-964, 966-967, 1026, 1029, 1073, 1076-1077, 1079, 1081, 1087-1088, 1095, 1099, 1106, 1110-1112, 1114-1117, 1119-1120, 1122-1123, 1126-1127 | 1461-1462, 1464-1466, 1468-1470, 1473, 1475-1477, 1480-1481, 1560, 1567, 1570-1571, 1574, 1765-1766, 1769-1770, 1773, 1820, 1824, 1828-1829, 1831-1835, 1837-1839, 1841, 1843, 1847, 1849-1850, 1852, 1854-1855, 1858-1859, 1906-1907, 1910, 1913-1914, 1917-1918, 1921, 1929, 1965-1970, 1972-1973, 1999-2000, 2003, 2007-2008, 2010-2012, 2014-2015, 2018-2019, 2022, 2026, 2068-2069, 2071-2073, 2119, 2122-2124, 2126-2131, 2134-2135, 2140-2143, 2153, 2157, 2160-2161, 2163-2164, 2166, 2169-2170, 2173, 2175, 2178, 2181, 2185-2186, 2188-2189, 2192, 2194-2196, 2222-2223, 2225-2226, 2228-2230, 2252, 2255, 2257-2258, 2262, 2279-2280, 2282-2284, 2292, 2295-2296, 2313-2314, 2317-2318, 2320, 2323-2324, 2328, 2330-2332, 2334, 2342-2343, 2345-2357 |
| **NCKAP1_iso2_-WAVE1** | 654 | 2550 |
| **NCKAP1_iso2_-ABI2** | 9-10, 12, 15-16, 18-19, 23, 30, 94-95, 98, 101, 105, 108, 112, 178-180, 464-465, 468, 491, 494-495, 498, 502, 586, 588-589, 591-592, 594, 606, 610, 705-709, 711-713, 715-716, 719, 760, 764-766, 768, 940, 943-944, 947, 951 | 2803, 2806-2807, 2810, 2813-2814, 2817, 2831-2840, 2842, 2845-2846, 2848-2858, 2860-2862, 2864-2869 |

**4. References:**

Bardoni B, Abekhoukh S. CYFIP family proteins between autism and intellectual disability: links with Fragile X syndrome. *Frontiers in Cellular Neuroscience* 8, 2014. https://doi.org/10.3389/fncel.2014.00081

Berjanskii M, Zhou J, Liang Y, Lin G, Wishart DS. Resolution-by-proxy: a simple measure for assessing and comparing the overall quality of NMR protein structures. *Journal of Biomolecular NMR* 53, 167-80, 2012. https://doi.org/10.1007/s10858-012-9637-2

Chen Z, Borek D, Padrick SB, Gomez TS, Metlagel Z, Ismail AM, Umetani J, Billadeau DD, Otwinowski Z, Rosen MK. Structure and control of the actin regulatory WAVE complex. *Nature* 468, 533-8, 2010. https://doi.org/10.1038/nature09623

Dehouck Y, Kwasigroch JM, Rooman M, Gilis D. BeAtMuSiC: prediction of changes in protein–protein binding affinity on mutations. *Nucleic Acids Research* 41, W333-W9, 2013. https://doi.org/10.1093/nar/gkt450

Ding B, Yang S, Schaks M, Liu Y, Brown AJ, Rottner K, Chowdhury S, Chen B. Structures reveal a key mechanism of WAVE regulatory complex activation by Rac1 GTPase. *Nature Communications* 13, 5444, 2022. <https://doi.org/10.1038/s41467-022-33174-3>

Edgar RC. MUSCLE: multiple sequence alignment with high accuracy and high throughput. *Nucleic Acids Research* 32, 1792-1797, 2004. https://doi.org/10.1093/nar/gkh340

Firth HV, Richards SM, Bevan AP, Clayton S, Corpas M, Rajan D, Vooren SV, Moreau Y, Pettett RM, Carter NP. DECIPHER: Database of Chromosomal Imbalance and Phenotype in Humans Using Ensembl Resources. *The American Journal of Human Genetics* 84, 524-33, 2009. https://doi.org/10.1016/j.ajhg.2009.03.010

Fu JM, Satterstrom FK, Peng M, Brand H, Collins RL, Dong S, Wamsley B, Klei L, Wang L, Hao SP*, et al.* Rare coding variation provides insight into the genetic architecture and phenotypic context of autism. *Nature Genetics* 54, 1320-31, 2022. https://doi.org/10.1038/s41588-022-01104-0

Guo H, Zhang Q, Dai R, Yu B, Hoekzema K, Tan J, Tan S, Jia X, Chung WK, Hernan R*, et al.* NCKAP1 Disruptive Variants Lead to a Neurodevelopmental Disorder with Core Features of Autism. *The American Journal of Human Genetics* 107, 963-76, 2020. https://doi.org/10.1016/j.ajhg.2020.10.002

Li D, Choque Olsson N, Becker M, Arora A, Jiao H, Norgren N, Jonsson U, Bölte S, Tammimies K. Rare variants in the outcome of social skills group training for autism. *Autism Research* 15, 434-46, 2022. https://doi.org/10.1002/aur.2666

Nakashima M, Kato M, Aoto K, Shiina M, Belal H, Mukaida S, Kumada S, Sato A, Zerem A, Lerman-Sagie T*, et al.* De novo hotspot variants in CYFIP2 cause early-onset epileptic encephalopathy. *Annals of Neurology* 83, 794-806, 2018. https://doi.org/10.1002/ana.25208

Pires DEV, Ascher DB, Blundell TL. mCSM: predicting the effects of mutations in proteins using graph-based signatures. *Bioinformatics* 30, 335-42, 2013. <https://doi.org/10.1093/bioinformatics/btt691>

Schaks M, Reinke M, Witke W, Rottner K. Molecular Dissection of Neurodevelopmental Disorder-Causing Mutations in CYFIP2. *Cells* 9, 1355, 2020. https://doi.org/10.3390/cells9061355

Schaks M, Singh SP, Kage F, Thomason P, Klünemann T, Steffen A, Blankenfeldt W, Stradal TE, Insall RH, Rottner K. Distinct Interaction Sites of Rac GTPase with WAVE Regulatory Complex Have Non-redundant Functions in Vivo. *Current Biology* 28, 3674-84.e6, 2018. <https://doi.org/10.1016/j.cub.2018.10.002>

Schrödinger, L., DeLano, W. PyMOL. Retrieved from http://www.pymol.org/pymol

Schymkowitz J, Borg J, Stricher F, Nys R, Rousseau F, Serrano L. The FoldX web server: an online force field. *Nucleic Acids Research* 33, W382-W8, 2005. https://doi.org/10.1093/nar/gki387

Sheth F, Shah J, Jain D, Shah S, Patel H, Patel K, Solanki DI, Iyer AS, Menghani B, Mhatre P*, et al.* Comparative yield of molecular diagnostic algorithms for autism spectrum disorder diagnosis in India: evidence supporting whole exome sequencing as first tier test. *BMC Neurology* 23, 292, 2023. https://doi.org/10.1186/s12883-023-03341-0

Singh A, Copeland MM, Kundrotas PJ, Vakser IA. GRAMM Web Server for Protein Docking. In: Gore M, Jagtap UB (eds). *Computational Drug Discovery and Design*. Pp 101-12. Springer US, New York, NY, 2024. https://doi.org/10.1007/978-1-0716-3441-7_5

Takenawa T, Suetsugu S. The WASP–WAVE protein network: connecting the membrane to the cytoskeleton. *Nature Reviews Molecular Cell Biology* 8, 37-48, 2007. https://doi.org/10.1038/nrm2069

Wang T, Hoekzema K, Vecchio D, Wu H, Sulovari A, Coe BP, Gillentine MA, Wilfert AB, Perez-Jurado LA, Kvarnung M*, et al.* Large-scale targeted sequencing identifies risk genes for neurodevelopmental disorders. *Nature Communications* 11, 4932, 2020. <https://doi.org/10.1038/s41467-020-18723-y>

Waterhouse A, Bertoni M, Bienert S, Studer G, Tauriello G, Gumienny R, Heer FT, de Beer TA P, Rempfer C, Bordoli L*, et al.* SWISS-MODEL: homology modelling of protein structures and complexes. *Nucleic Acids Research* 46, W296-W303, 2018. <https://doi.org/10.1093/nar/gky427>

Webb B, Sali A. Protein Structure Modeling with MODELLER. In: Chen YW, Yiu C-PB (eds). *Structural Genomics: General Applications*. Pp 239-55. Springer US, New York, NY, 2021. <https://doi.org/10.1007/978-1-0716-0892-0_14>

Zhou X, Feliciano P, Shu C, Wang T, Astrovskaya I, Hall JB, Obiajulu JU, Wright JR, Murali SC, Xu SX*, et al.* Arf GTPase activates the WAVE regulatory complex through a distinct binding site. *Science Advances* 8, 50, 2022. https://doi.org/ 10.1126/sciadv.add1412.

Zweier M, Begemann A, McWalter K, Cho MT, Abela L, Banka S, Behring B, Berger A, Brown CW, Carneiro M*, et al.* Spatially clustering de novo variants in CYFIP2, encoding the cytoplasmic FMRP interacting protein 2, cause intellectual disability and seizures. *European Journal of Human Genetics* 27, 747-59, 2019. https://doi.org/10.1038/s41431-018-0331-z
